# Targeting CCR2^+^ macrophages with BET inhibitor overcomes adaptive resistance to anti-VEGF therapy in ovarian cancer

**DOI:** 10.1007/s00432-021-03885-z

**Published:** 2022-01-30

**Authors:** Yutuan Wu, Nicholas B. Jennings, Yunjie Sun, Santosh K. Dasari, Emine Bayraktar, Sara Corvigno, Elaine Stur, Deanna Glassman, Lingegowda S. Mangala, Adrian Lankenau Ahumada, Shannon N. Westin, Anil K. Sood, Wei Hu

**Affiliations:** 1 Department of Gynecologic Oncology and Reproductive Medicine, The University of Texas MD Anderson Cancer Center, 1155 Herman Pressler Boulevard, Unit 1362, Houston, TX 77030 USA; 2 Department of Gynecologic Oncology, Fudan University Shanghai Cancer Center, Shanghai, People’s Republic of China; 3 Department of Oncology, Shanghai Medical College, Fudan University, Shanghai, People’s Republic of China; 4 Department of Cancer Biology, The University of Texas MD Anderson Cancer Center, Houston, TX USA; 5 Center for RNA Interference and Non-Coding RNA, The University of Texas MD Anderson Cancer Center, Houston, TX USA

**Keywords:** BET inhibitor, CCR2, Macrophages, Anti-VEGF antibody (AVA) therapy, Ovarian cancer

## Abstract

**Purpose:**

Tumor-associated macrophages (TAMs) are known to contribute to adaptive resistance to anti-vascular endothelial growth factor (VEGF) antibody (AVA) therapy in ovarian cancer. BET (bromodomain and extra-terminal domain) inhibitors (BETi) may have unique roles in targeting TAMs. Our objective was to examine the effects of BETi on TAMs, especially in the context of enhancing the efficacy of AVA therapy.

**Methods:**

We conducted a series of in vitro (MTT assay, apoptosis, flow cytometry, and RNA sequencing) and in vivo (xenograft ovarian cancer model) experiments to determine the biological effects of BETi combined with AVA in ovarian cancer. For statistical analysis, a two-tailed Student’s *t* test (equal variance) or ANOVA was used for multiple groups’ comparison, and *p* < 0.05 was considered significant.

**Results:**

BETi resulted in a dose-dependent decrease in cell viability and induced apoptosis (*p* < 0.01) in ovarian cancer cells (SKOV3ip1, OVCAR5, and OVCAR8). Treatment with BETi significantly increased apoptosis in THP-1 monocytes and macrophages (PMA-differentiated THP-1; *p* < 0.01). Furthermore, BETi selectively induced greater apoptosis in M2-like macrophages (PMA and IL-4, IL-13-differentiated THP-1) (31.3%-36.1%) than in M1-like macrophages (PMA and LPS-differentiated THP-1) (12.4%-18.5%) (*p* < 0.01). Flow cytometry revealed that the percentage of M1-like macrophages (CD68^+^/CD80^+^) was significantly increased after treatment with low-dose BETi (ABBV-075 0.1 µM; *p* < 0.05), whereas the percentage of CD68^+^/CCR2^+^ macrophages was significantly decreased (*p* < 0.001); these findings suggest that BETi may selectively inhibit the survival of CCR2^+^ macrophages and re-polarize the macrophages into an M1-like phenotype. RNA-seq analysis revealed that BETi selectively targeted macrophage infiltration-related cytokines/chemokines in ovarian cancer (adjusted *p* < 0.05 and Log2 fold change ≥ 1.5). Finally, using in vivo ovarian cancer models, compared with control or monotherapy, the combination of BETi (ABBV-075) and bevacizumab resulted in greater inhibition of tumor growth and macrophage infiltration (*p* < 0.05) and longer survival of tumor-bearing mice (*p* < 0.001).

**Conclusions:**

Our findings indicate a previously unrecognized role for BETi in selectively targeting CCR2^+^ TAMs and enhancing the efficacy of AVA therapy in ovarian cancer.

**Supplementary Information:**

The online version contains supplementary material available at 10.1007/s00432-021-03885-z.

## Introduction

Angiogenesis is known to play an important role in tumor growth and progression. Anti-angiogenesis approaches, particularly anti-vascular endothelial growth factor (VEGF) therapy, have been approved by the U.S. Food and Drug Administration (FDA) for many cancers (Ferrara and Adamis 2016). Bevacizumab, a neutralizing anti-VEGF antibody (AVA), was the first AVA therapeutics to be approved by the FDA for ovarian cancer (2014) (Ferrara and Adamis 2016). Several phase III trials of bevacizumab in combination with chemotherapy have demonstrated significant improvement in progression free survival, but without significant improvement in overall survival in patients with ovarian cancer (Aghajanian et al. 2012; Burger et al. 2011; Coleman et al. 2017; Perren et al. 2011; Pujade-Lauraine et al. 2014). The rapid emergence of adaptive resistance results in limited durability of AVA therapy for cancer, and the mechanism of resistance to AVA therapy remains largely undefined (Dalton et al. 2017; Tewari et al. 2019). Many factors contribute to adaptive resistance to AVA therapy, including host-mediated resistance and tumor-mediated resistance (Ma et al. 2018). Our previous studies demonstrated that macrophages play an important role in promoting the development of resistance to AVA therapy (Dalton et al. 2017; Lyons et al. 2017). Importantly, the ratio of high M2-like/M1-like macrophages has been reported to be associated with AVA resistance, and M2 polarization of macrophages may contribute to AVA resistance (Castro et al. 2017). Therefore, targeting macrophages could be a potential novel strategy for overcoming resistance to AVA therapy in ovarian cancer.

In the tumor microenvironment, polarization of tumor-associated macrophages (TAM) translates into heterogeneous phenotypes with different functions. TAMs with protumoral potential are referred to as M2-like macrophages; they have immunosuppressive effects that contribute to angiogenesis, tumor growth, and metastasis (Cassetta and Pollard 2018). Macrophages can also be tumoricidal and inhibit tumor growth by activating immune responses; these are known as M1-like macrophages (Cassetta and Pollard 2018). According to the different properties and plasticity of TAMs, targeting macrophage polarization may be a novel therapeutic approach that could rebalance the microenvironmental immune infiltrates from protumoral M2-like macrophages to tumoricidal M1-like macrophages (Cassetta and Pollard 2018).

Expansion of TAMs is often regulated by recruitment of monocytes through the C–C motif chemokine ligand 2 (CCL2)/C–C motif chemokine receptor 2 (CCR2) axis. CCL2 is responsible for the recruitment and migration of CCR2^+^ inflammatory monocytes to tumors where they become immunosuppressive TAMs. CCL2/CCR2 signaling has been correlated with poor prognosis and metastatic disease (Qian et al. 2011). CCR2 blockade has been shown to reduce TAM infiltrates and increase antitumor immune response (Nywening et al. 2016). Importantly, a clinical trial with a CCR2 inhibitor resulted in better overall survival in patients with locally advanced/metastatic pancreatic cancer, and clinical response was associated with lower peripheral blood monocyte counts at baseline (Linehan et al. 2018). Notably, our previous study showed that the interaction of microseminoprotein (MSMP), a CCR2 ligand, with CCR2 plays an important role in promoting adaptive resistance to AVA in ovarian cancer models (Mitamura et al. 2018). However, the underlying mechanisms by which MSMP/CCR2 mediates adaptive resistance are not fully understood (Mitamura et al. 2018; Pei et al. 2014).

In this study, we sought to investigate the role of MSMP/CCR2 signaling in macrophage polarization and the effects of targeting CCR2^+^ macrophages with bromodomain and extra-terminal domain (BET) inhibitors (BETi) in ovarian cancer, especially in the context of enhancing the efficacy of AVA therapy. Our findings suggested a previously unrecognized role for BETi in selectively targeting CCR2^+^ macrophages and enhancing the efficacy of AVA therapy in ovarian cancer.

## Materials and methods

### Reagents

BETi (ABBV-075, S8400; and ABBV-744, S8723) were purchased from Selleckchem (Houston, TX). CPI 203 (SML1212), phorbol 12-myristate 13-acetate (PMA) (P1585), and lipopolysaccharides (LPS) (L4391) were purchased from Sigma-Aldrich (St. Louis, MO). Bevacizumab (Genentech, South San Francisco, CA) was obtained from the MD Anderson Pharmacy and used for in vivo treatment. Interleukin 4 (IL-4) (200-04) and interleukin 13 (IL-13) (200-13) were purchased from PEPROTECH (Cranbury, NJ) and used for M2-like macrophage differentiation. For Western blot analysis, we used primary antibodies of anti-CCR2 (1:500, 711255, RRID: AB_2633142, Thermo Scientific, Waltham, MA), and anti-*β*-actin (1:1000, A5441, RRID: AB_476744, Sigma-Aldrich, St. Louis, MO). For immunohistochemical (IHC) staining, we used the following primary antibodies: anti-F4/80 (1:100, MCA497G, RRID: AB_872005, Bio-Rad, Hercules, CA), anti-Arginase-1 (ARG1) (1:200, 93668, RRID: AB_2800207, Cell Signaling, Danvers, MA), anti-Ki67 (1:100, RB9043P, RRID: AB_149874, Thermo Scientific, Waltham, MA), anti-CD31 (1:800, 553370, RRID: AB_394816, BD Biosciences, Franklin Lake, NJ), and anti-Cleaved PARP (Asp214) (D64E10) (1:50, 5625, RRID: AB_10699459, Cell Signaling, Danvers, MA).

### Cell lines and tissue cultures

Human monocytic THP-1 (RRID: CVCL_0006) cells and ovarian cancer cells (SKOV3ip1 [RRID: CVCL_0532], OVCAR5 [RRID: CVCL_1628], and OVCAR8 [RRID: CVCL_1629] cell lines) were obtained from the American Type Culture Collection (ATCC) and the University of Texas MD Anderson Cancer Center Characterized Cell Line Core Facility. All cell lines were validated by STR fingerprinting in the Characterized Cell Line Core Facility. Cell lines were screened for mycoplasma and were grown in RPMI-1640 medium (HyClone, Logan, UT) supplemented with 10% fetal bovine serum (Sigma-Aldrich, St. Louis, MO) and 1% penicillin and streptomycin at 37 °C in a 5% CO_2_ humidified atmosphere. The THP-1 cells were differentiated into macrophages by adding 30 ng/ml PMA for 24 h. After that, macrophages were polarized into M1-like macrophages by incubating with 15 ng/ml LPS for 48 h, and M2-like macrophages polarization was induced by incubating with 25 ng/ml IL-4 and 25 ng/ml IL-13 for 48 h (Smith et al. 2015).

### Cell viability and colony formation assays

To detect the cytotoxic effects of BETi on cell viability, ovarian cancer cells (SKOV3ip1, OVCAR5, and OVCAR8) were seeded in 96-well plates in single-cell suspensions at an initial density of 3000 cells per well with quadruplicates. After cells were seeded overnight, culture media were replaced with 100 µl of culture media with specific concentrations of BETi (ABBV-075, ABBV-744, or CPI 203) at 0, 0.01, 0.1, 1.0, 5.0, 10.0, and 20.0 µmol/l for 48, 72 and 96 h. After treatment, 50 µl of 1.5 mg/ml MTT solution (J19265, Affymetrix, Santa Clara, CA) was added to each well of the 96-well plates. After incubation for 2 h at 37 °C, the MTT mixed solution was gently removed from each well. Then, 100 µl of DMSO was added to each well. After the MTT byproduct metabolized formazan was completely dissolved during incubation for 30 min, an absorbance value at 540 nm was detected by a microplate spectrophotometer reader. Each experiment was repeated three times.

For colony formation assay, ovarian cancer cells (SKOV3ip1, OVCAR5, and OVCAR8) were seeded in 6-well plates at an initial density of 1000 cells per well with quadruplicates. After seeding overnight, cells were cultured with fresh media containing vehicle control or BETi (0.1 and 1.0 µmol/l) for 7–10 days. After that, the cells were washed with PBS, fixed with 4% paraformaldehyde for 15 min; then, cells were stained with 0.1% crystal violet solution for 15 min at room temperature. Finally, the cells were washed with PBS and left to dry at room temperature (Villar-Prados et al. 2019).

### Flow cytometry assay

Apoptosis was detected using a FITC Annexin V Apoptosis Detection Kit (556547, RRID: AB_2869082, BD Bioscience, Franklin Lake, NJ). After 48 h of treatment, cells were washed with PBS and trypsinized with 0.25% trypsin/EDTA at 37 °C and then re-suspended in 1 × binding buffer (1 × 10^6^ cells/ml). Then, 100 µl of the solution (1 × 10^5^ cells) was transferred to a flow tube for flow cytometry. Next, 5 µl of FITC Annexin V and 5 µl of PI were added to each tube. We used several controls including unstained cells (negative), cells stained with FITC Annexin V alone, and cells stained with PI alone. The cells were gently vortexed and incubated for 15 min at room temperature (25 °C) in the dark. 400 µl of 1 × binding buffer was added to each tube and then analyzed by flow cytometry within 1 h.

To analyze cell populations of interest, flow cytometry was performed using specific antibodies. For cell surface markers, macrophage suspensions were stained with conjugated fluorescent antibodies of anti-CD206 (321124, RRID: AB_10933248, BioLegend, San Diego, CA), anti-CD80 (305216, RRID: AB_528875, BioLegend, San Diego, CA), and anti-CCR2 (357206, RRID: AB_2562059, BioLegend, San Diego, CA), and then incubated on ice for 15–20 min in the dark. For intracellular markers, cells were fixed by 4% paraformaldehyde and permeabilized by 0.8% triton X-100 and then stained with anti-CD68 antibody (333828, RRID: AB_2800882, BioLegend, San Diego, CA) for 20 min in the dark at room temperature. Cells were incubated with Fc receptor blocking solution (422301, RRID: AB_2818986, BioLegend, San Diego, CA) for 5–10 min at room temperature to reduce nonspecific immunofluorescence staining before primary antibody staining. Fluorescence compensations were performed by UltraComp eBeads (01–2222-42, Invitrogen, Waltham, MA) by single staining of conjugated fluorescent antibody. If cell surface markers and intracellular markers were stained in the same panel, cell surface marker staining was first performed, and cells were then fixed with fixation solution to stain intracellular markers. The SYTOX (S7020, Invitrogen, Waltham, MA) or LIVE/DEAD fixable green dead cell stain kit (L34969, Invitrogen, Waltham, MA) was used to determine the viability of cells before fixation and permeabilization. Flow cytometry analyses were performed on an LSRFortessa X-20 flow cytometer (BD Biosciences, Franklin Lake, NJ) with BD FACSDiva 8.0.1 software.

### Quantitative real-time PCR (qRT-PCR)

Total mRNAs were extracted from ovarian cancer cells (SKOV3, OVCAR5, and OVCAR8) and macrophages at 24 or 48 h after treatment with BETi using a Direct-zol RNA MiniPrep Kit (11-331, Zymo Research, Irvine, CA) according to the manufacturer’s protocol. Quantity and quality were detected by NanoDrop (Thermo Scientific, Waltham, MA). After that, mRNAs were reverse transcribed into cDNA using the Verso cDNA Synthesis Kit (AB1453B, Thermo Scientific, Waltham, MA). Then, real-time PCR amplification for specific genes was performed as previously described (Villar-Prados et al. 2019). 18S rRNA expression was used as an endogenous control. The primers were synthesized by Sigma-Genosys, and sequences are listed in supplementary table 1.

### Western blotting

Total protein was extracted using modified RIPA buffer with protease and phosphatase inhibitors. The protein concentration was detected by BCA Protein Assay Reagent (23228, Thermo Scientific, Waltham, MA). Protein expression was detected by Western blotting of SDS-PAGE separation with use of the primary antibodies of anti-CCR2. The primary antibody was incubated overnight at 4 °C and then interacted with corresponding horseradish peroxidase-linked whole secondary antibodies. The membranes were exposed using a chemiluminescence assay by Western Lightning Plus ECL Kit (NEL104001EA, PerkinElmer, Waltham, MA). The bands were quantified by densitometry using Image-J. β-actin was used for sample loading control (Villar-Prados et al. 2019).

### In vivo experiments

For in vivo studies, female athymic (NCr-nude) mice (5–8 weeks old) were purchased from Taconic Biosciences and housed in specific pathogen-free conditions. Mice were cared for according to the guidelines of the American Association for Accreditation for Laboratory Animal Care and the U.S. Public Health Service Policy on Humane Care and Use of Laboratory Animals. The animal experimental protocols were approved by the Institutional Animal Care and Use Committee at MD Anderson Cancer Center. To establish the ovarian cancer xenograft models, luciferase-labeled SKOV3ip1 ovarian cancer cells were cultured to reach about 60–80% confluence, and 1 × 10^6^ cells were intraperitoneally inoculated into the right side of mice. One week after cell inoculation in the co-treatment model, mice received vehicle control, ABBV-075 alone, bevacizumab alone, or combination therapy. For the resistance model, three weeks after cell injection, mice were injected with 200 μl of 14.3 mg/ml luciferin (LUCK-1G, Gold Bio, St Louis, MO) and then examined by the Xenogen in vivo imaging system (IVIS) (2 × 10^6^ photons/s/cm^2^/sr). IVIS imaging was conducted weekly. Mice without tumors were removed from the experiment. Tumor-bearing mice were randomized to the vehicle control group, ABBV-075 group, and bevacizumab group to receive the proposed treatments. After two weeks of treatment, the mice that had received bevacizumab were separated into bevacizumab-sensitive or bevacizumab-resistant groups according to the IVIS imaging signal (Dalton et al. 2017). The mice in the bevacizumab-sensitive group were euthanized and the mice in the bevacizumab-resistant group were randomized to receive bevacizumab alone or bevacizumab combined with ABBV-075. Mice were monitored daily for adverse effects and measured for body weight weekly. Mice were euthanized once they became moribund due to tumor burden. Mouse body weight, tumor weight, tumor nodules, and distribution of tumors were recorded at the death of each mouse. For the survival model, survival time was calculated for each mouse according to the time the mouse became moribund and was euthanized. Tissue specimens were snap frozen for later protein and RNA analysis, frozen slide preparation or fixed in formalin for paraffin embedding. All investigators who performed the necropsies were blinded to the treatment group assignments. The animal models used here have been described previously (Dalton et al. 2017; Mitamura et al. 2018; Villar-Prados et al. 2019).

### IHC staining

Formalin-fixed paraffin-embedded slides were used for staining with Ki67, F4/80, ARG1, or cleaved PARP antibodies. Frozen slides were used for IHC staining with CD31 antibody. Paraffin slides were processed by deparaffinization/rehydration and antigen retrieval; frozen slides were processed by cold acetone fixation; all samples were then followed by endogenous peroxidase blocking with 3% hydrogen peroxide, protein blocking with 4% fish gelatin, and incubated with primary antibody overnight, then incubated with peroxidase-conjugated goat anti-rabbit or anti-mouse secondary antibody for 1 h at room temperature. DAB staining was monitored microscopically. Finally, the slides were counterstained with Gill’s hematoxylin #3. For IHC analysis, five random fields at × 200 magnification were taken per slide (Villar-Prados et al. 2019).

### RNA sequencing and bioinformatics analysis

Total mRNA was extracted from the SKOV3 cells and from M1-like (PMA, LPS) and M2-like (PMA, IL-4 + IL-13) macrophages after 24 h of treatment (ABBV-075, 1.0 µM for SKOV3, 0.01 µM for M1 and M2) using Direct-zol RNA MiniPrep Kit (11-331, Zymo Research, Irvine, CA). RNA samples were submitted to Novogene Co., Ltd. (Sacramento, CA) for RNA-seq analysis. Briefly, sample quality control (QC) was done to meet the sequencing criteria and ensure the reliability of results. The single-stranded mRNAs were selectively enriched and converted to cDNA for library preparation. Sequencing was then performed using PE150 strategy based on the Illumina NovaSeq platform. The sequencing data were checked for quality eligibility before bioinformatics analysis. Gene expression level was estimated by the read count of sequencing, which is the abundance of transcripts that mapped to the genome or exon. HTSeq v0.6.1 was used to count the read numbers of genes. The fragments per kilobase of transcript sequence per million (FPKM) value of each gene was calculated on the basis of gene length and read counts mapped to this gene. The FPKM value was used for quantification of gene expression level. Differential gene expression analysis between control and treatment group was performed by DESeq2 R package (2_1.6.3). A differentially expressed gene was defined as having a concurrent adjusted *p* < 0.05 and Log2 fold change ≥ 1.5. Heatmaps of genes were performed by GraphPad Prism 8 using z-scores. The workflow of RNA sequencing and bioinformatics analysis were performed by Novogene Co., Ltd.

We utilized the Database of Immune Cell Expression (DICE) (https://dice-database.org/) to explore immune cell gene expression (Schmiedel et al. 2018). In addition, we explored the associations between immune infiltrates and genetic or clinical features for ovarian cancer in The Cancer Genome Atlas (TCGA) cohort by database of Tumor Immune Estimation Resource 2.0 (TIMER2.0) (http://cistrome.org/TIMER/), all the algorithms were described by TIMER2.0 (Li et al. 2020).

### Statistical analysis

Differences between two groups were evaluated by the Student's *t* test or with Welch’s correction according to data distribution and variance homogeneity, whereas one-way ANOVA was used for multiple groups’ comparison. For survival experiments, Kaplan–Meier analysis and the log-rank test were performed to evaluate the differences in survival. All statistical analyses were conducted by GraphPad Prism 8. The *p* values were two-tailed and statistical significance was defined as *p* < 0.05. All results were presented as the mean ± SEM or SD.

## Results

### BETi decreases CCR2 and MSMP expression and macrophage infiltration in ovarian cancer

Given the potential role of MSMP/CCR2 in TAM recruitment in the tumor microenvironment, we analyzed CCR2 expression across immune cells with use of the DICE database (Schmiedel et al. 2018). Our analysis revealed that CCR2 expression was significantly higher in classical monocytes than in non-classical monocytes (*p* < 0.001) (Supplementary Fig. 1A). Since MSMP is an important ligand for CCR2, we further analyzed MSMP expression from the TCGA cohort by TIMER2.0 (Li et al. 2020). We found that MSMP expression was relatively high in ovarian cancer (Supplementary Fig. 1B). We then evaluated the correlation between CCR2/MSMP expression and macrophage infiltration in ovarian cancer and found that CCR2 expression was significantly correlated with M1-like and M2-like macrophage infiltration (*p* < 0.001), whereas MSMP expression significantly correlated with only M2-like macrophage infiltration (*p* < 0.01) (Supplementary Fig. 1C, D). Collectively, the bioinformatics analysis of TCGA data indicated that expression of CCR2 and its ligand MSMP was related with macrophage infiltration in ovarian cancer.

**Fig. 1:**
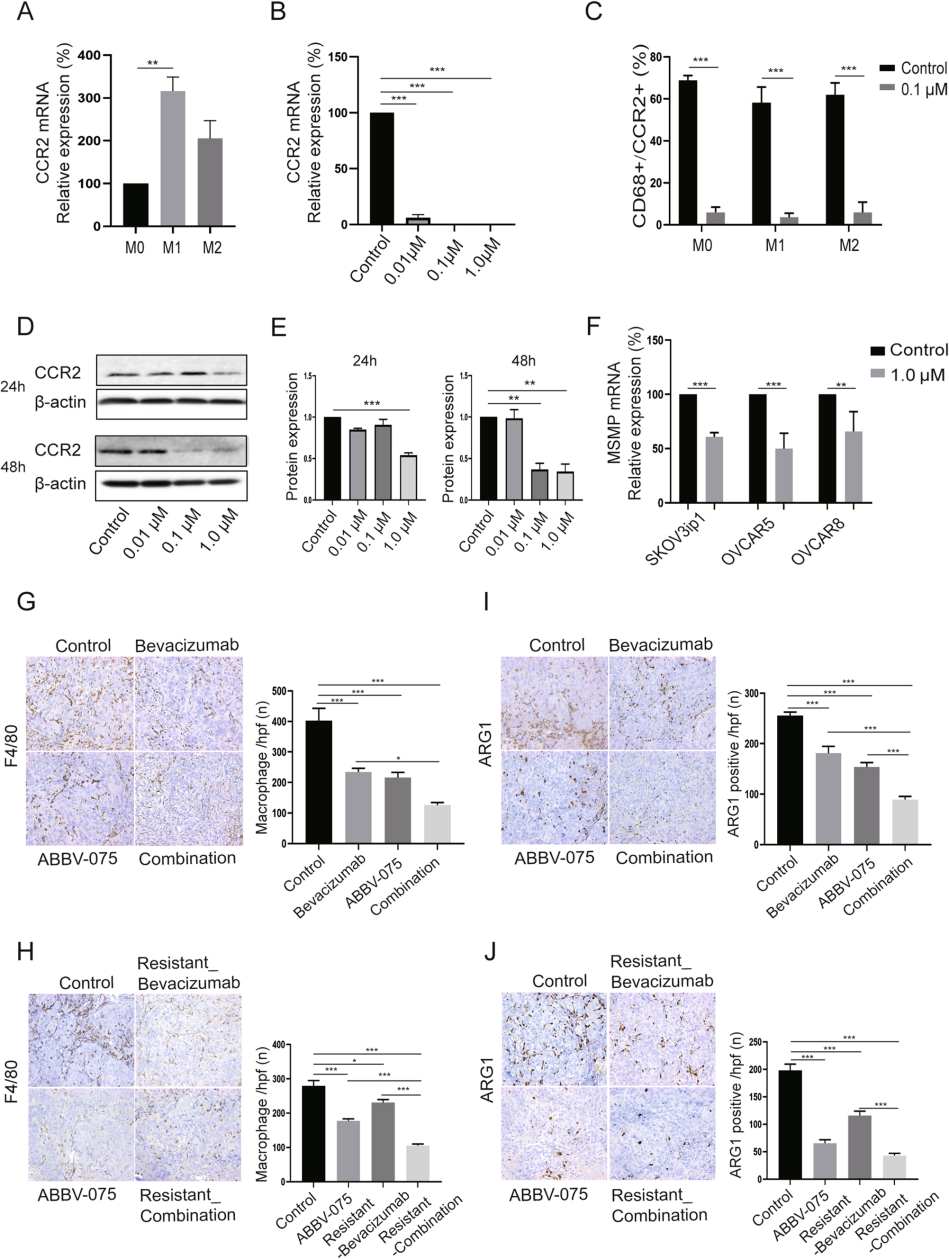
BETi decreased macrophage infiltration in ovarian tumor by targeting MSMP/CCR2 signaling. **A** qRT-PCR analysis of baseline level of CCR2 expression in different macrophage subsets: M0 (PMA), M1 (PMA, LPS), and M2 (PMA, IL-4 + IL-13). **B** qRT-PCR analysis of CCR2 expression in macrophages after ABBV-075 treatment for 24 h. **C** Flow cytometry analysis of CD68^+^/CCR2^+^ macrophages after 0.1 µM ABBV-075 treatment for 48 h. **D, E** Western blot of CCR2 expression in macrophages after ABBV-075 treatment for 24 or 48 h; bar graphs show the quantification of band intensity (normalized to β-actin) relative to control intensity. **F** qRT-PCR analysis of MSMP expression in ovarian cancer cell lines (SKOV3ip1, OVCAR5 and OVCAR8) after 1.0 µM ABBV-075 treatment for 24 h. **G** IHC stain analysis of macrophage infiltration (F4/80) in SKOV3ip1 ovarian cancer co-treatment model (× 200 magnification). **H** IHC stain analysis of macrophage infiltration (F4/80) in SKOV3ip1 ovarian cancer adaptive resistance model (× 200 magnification). **I** IHC stain analysis of M2-like macrophages (ARG1) in SKOV3ip1 ovarian cancer co-treatment model (× 200 magnification). **J** IHC stain analysis of M2-like macrophages (ARG1) in SKOV3ip1 ovarian cancer adaptive resistance model (× 200 magnification). Data represent the mean of three in vitro experiments for qRT-PCR and Western blot. Bar graphs: mean ± SEM. *p < 0.05, **p < 0.01, and *** *p* < 0.001

BETi have been shown to bind with bromodomains, resulting in significant epigenetic transcriptional regulation. BETi may have unique roles in targeting TAMs and CCR2 since CCR2 has been reported to be a pharmacodynamic biomarker for clinical use of BETi (Yeh et al. 2017). To evaluate the effects of BETi on CCR2 and MSMP expression and macrophage infiltration, we examined the macrophage markers after BETi treatment in vitro and in vivo. We first confirmed that CCR2 was highly expressed in macrophages (M0: PMA-differentiated THP-1; M1: PMA and LPS-differentiated THP-1; M2: PMA and IL-4, IL-13-differentiated THP-1) (Fig. 1A). We then found that BETi significantly decreased CCR2 expression in macrophages, detected by qRT-PCR (Fig. 1B), flow cytometry (Fig. 1C), and Western blotting analysis (*p* < 0.01) (Fig. 1D, E). Given that MSMP has been identified as a novel CCR2 ligand and cancer-secreted protein, we then tested MSMP expression in ovarian cancer cells by qRT-PCR. MSMP expression was significantly decreased after BETi treatment (*p* < 0.01) (Fig. 1F).

Infiltration of M2-like macrophages in ovarian cancer is known to promote tumor progression and facilitate resistance to anticancer therapy. By IHC analysis of the tumor tissues from the SKOV3ip1 xenograft tumor co-treatment model and adaptive resistance model, we found that TAMs (F4/80 +) were significantly decreased in ovarian tumors after treatment with ABBV-075 and AVA (*p* < 0.05) (Fig. 1G, H). We further investigated the M2-like macrophages in ovarian tumor by IHC analysis for ARG1, a hallmark of alternatively activated macrophages; there was a significant decrease after ABBV-075 and AVA treatment (*p* < 0.001) (Fig. 1I, J).

### Cytotoxic effects of BETi on ovarian cancer cells

**Fig. 2:**
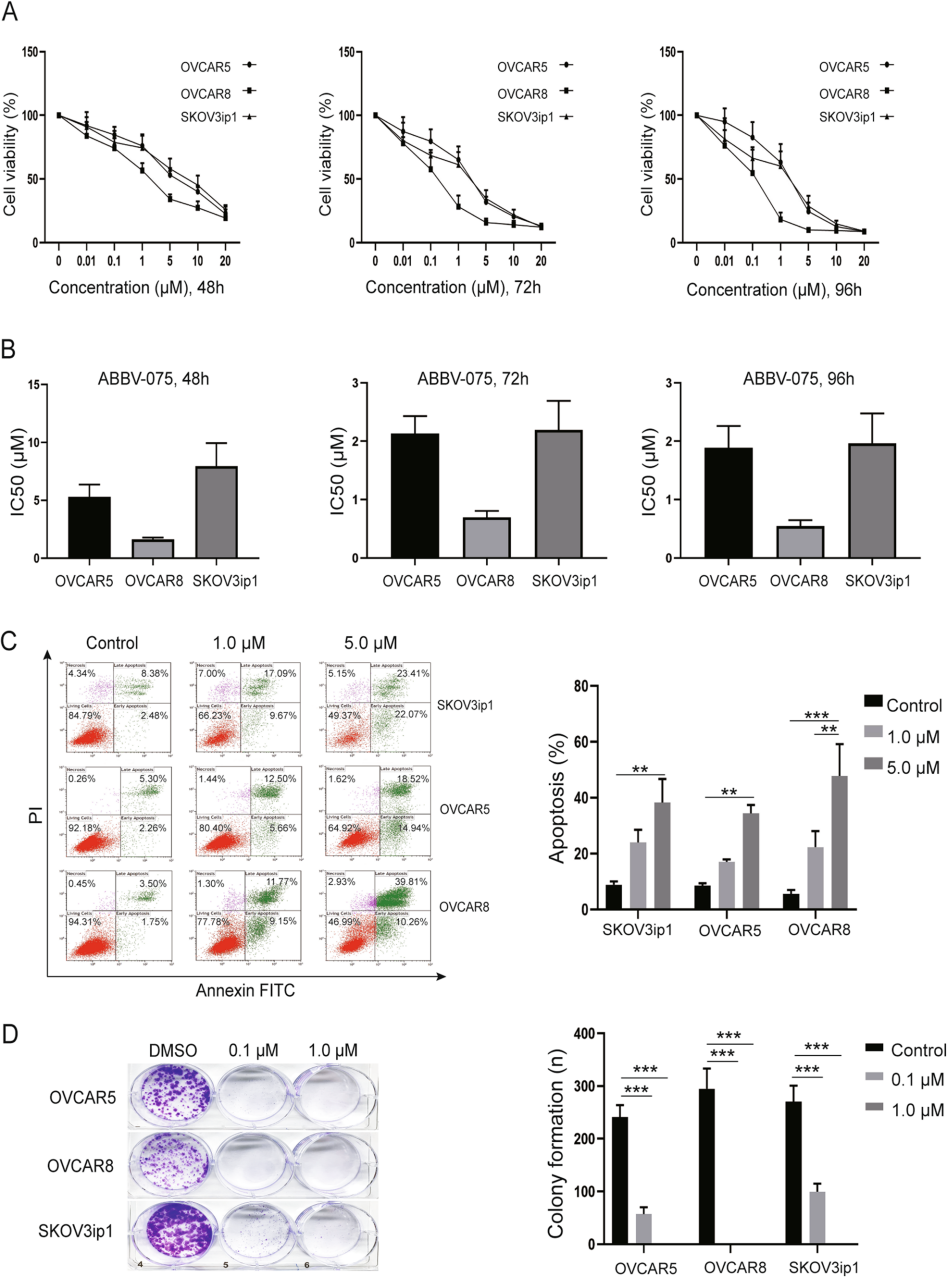
Antitumor effects of BETi on ovarian cancer cells. **A** MTT cell viability assay of ovarian cancer cells (SKOV3ip1, OVCAR5, and OVCAR8) after treatment with ABBV-075 for 48, 72, and 96 h. Experiments were performed in triplicate by independent assays. **B** IC50 values of ABBV-075 after for 48, 72, and 96 h of treatment in SKOV3ip1, OVCAR5, and OVCAR8 cells. **C** Flow cytometry analysis of apoptosis in SKOV3ip1, OVCAR5, and OVCAR8 cells after treatment with ABBV-075 for 48 h. Representative images (left) and graphs of results (right). Experiments were performed in triplicate by independent assays. **D** Effect of 7–10 days of ABBV-075 treatment on colony formation in SKOV3ip1, OVCAR5, and OVCAR8 cells. Experiments were performed in triplicate by independent assays. Bar graphs: mean ± SEM. *p < 0.05, ** *p* < 0.01, and ***p < 0.001

To identify the therapeutic effects of BETi on ovarian cancer cells, we evaluated the cytotoxic effects of various BETi (CPI203, ABBV-744, and ABBV-075). CPI203 binds directly to the BRD4 protein bromodomains, whereas ABBV-744 selectively targets the second (BD2) domain of BET family proteins; ABBV-075 targets the first (BD1) and the second (BD2) domain of BET family proteins. The MTT assay demonstrated significant dose-dependent inhibition of cell viability after treatment with ABBV-075, ABBV-744, and CPI203 for 48, 72 and 96 h (Fig. 2A, Supplementary Fig. 2A, B). The IC50 values of ABBV-075 and ABBV-744 in ovarian cancer cell lines ranged from 0.5 to 7.9 µM and from 2.6 to 10.3 µM, respectively, after treatment for 48, 72 and 96 h (Fig. 2B, Supplementary Fig. 2C). However, IC50 of CPI 203 was only achieved in OVCAR8 cells after treatment for 72 and 96 h (1.6 to 2.1 µM), but not achieved in other cell lines and time-points. Next, we examined the apoptotic effects of BETi on ovarian cancer cell lines by flow cytometry. ABBV-075 induced significantly higher apoptosis in all ovarian cancer cell lines compared with control treatment (*p* < 0.01) (Fig. 2C); CPI 203 induced significant apoptosis only in SKOV3ip1 cells, and ABBV-744 induced significant apoptosis in SKOV3ip1 and OVCAR8 cells (*p* < 0.01) (Supplementary Fig. 2D, E). In addition, colony formation assays showed a significant decrease in ovarian cancer cell growth by BETi treatment (*p* < 0.05); ABBV-075 treatment resulted in decreased colony formation (Fig. 2D), compared with other BETi (Supplementary Fig. 2F, G). We, therefore, focused on ABBV-075 for further in vitro and in vivo studies.

### BETi selectively induce apoptosis in M2-like macrophages and reprogram macrophages toward an M1-like phenotype

**Fig. 3:**
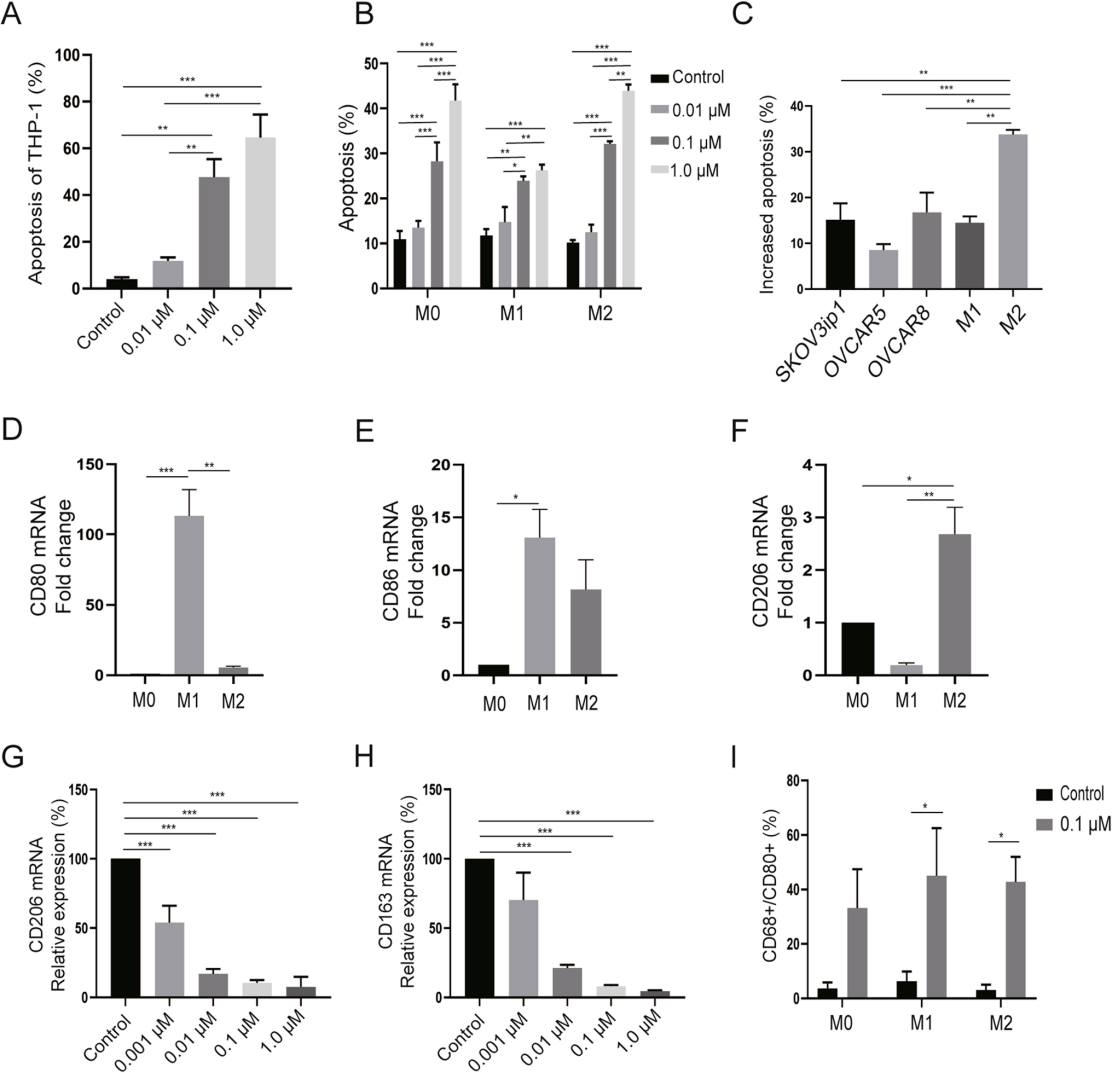
Effects of BETi on apoptosis and reprogramming in macrophages. **A, B** Flow cytometry analysis of apoptosis in THP-1 monocytes and M0 (PMA), M1 (PMA, LPS), and M2 (PMA, IL-4 + IL-13) macrophages after treatment with ABBV-075 for 48 h. Experiments were performed in triplicate by independent assays. **C** Flow cytometry analysis of increased apoptosis compared with controls (Increased apoptosis = treatment group (%)—control group (%)) in SKOV3ip1, OVCAR5, OVCAR8, M1 (PMA, LPS), and M2 (PMA, IL-4 + IL-13) macrophages after treatment with ABBV-075 (1.0 µM) for 48 h. **D, E** qRT-PCR analysis of baseline expression level of M1-like macrophage markers (CD80 and CD86) in M0 (PMA), M1 (PMA, LPS), and M2 (PMA, IL-4 + IL-13) macrophages (normalized to expression level in M0). **F** qRT-PCR analysis of baseline expression level of M2-like macrophage marker (CD206) in M0 (PMA), M1 (PMA, LPS), and M2 (PMA, IL-4 + IL-13) macrophages (normalized to expression level in M0). **G, H** Relative expression of M2-like macrophage markers (CD206 and CD163) after treatment with ABBV-075 for 24 h by qRT-PCR analysis. **I** Flow cytometry analysis of CD68^+^/CD80^+^ M1-like macrophage proportion after treatment with 0.1 µM ABBV-075 for 48 h. Experiments were performed in triplicate by independent assays. Bar graphs: mean ± SEM. *p < 0.05, **p < 0.01, and ***p < 0.001

To investigate whether BETi selectively target M1-like or M2-like macrophages, we assayed for apoptosis in THP-1 monocytes and macrophages after treatment with ABBV-075. By flow cytometry analysis, we found that ABBV-075 significantly increased apoptosis in monocytes/macrophages (*p* < 0.01) (Fig. 3A, B); more specifically ABBV-075 (1.0 µM) selectively induced more apoptosis in M2-like macrophages (31.3–36.1%) than in M1-like macrophages (12.4–18.5%) or in ovarian cancer cells (SKOV3ip1: 5.8–23.34%, OVCAR5: 4.86–10.6%, OVCAR8: 7.8–28.64%) (*p* < 0.01) (Fig. 3C).

To further investigate the reprogramming effects of BETi on macrophages, we first confirmed that CD80 and CD86 were expressed mostly in M1-like macrophages (PMA, LPS) (*p* < 0.05) (Fig. 3D, E), whereas CD206 was expressed primarily in M2-like macrophages (PMA, IL-4 + IL-13) (*p* < 0.05) (Fig. 3F). We then tested the effects of ABBV-075 on M2-like markers and found that expression of M2-like macrophage marker (CD206 and CD163) was significantly decreased after 24 h of treatment with ABBV-075, as detected by qRT-PCR (*p* < 0.001) (Fig. 3G, H). Furthermore, flow cytometry was used to detect the effects of ABBV-075 on macrophage reprogramming after 48 h of treatment at low concentrations (0.1 µM). Flow cytometry analysis showed an increase in CD80 expression in CD68^+^ macrophages after BETi treatment (*p* < 0.05) (Fig. 3I, Supplementary Fig. 3A). CD68^+^ macrophages were gated on live cells, and the M1 marker (CD80) was used to gate M1-like macrophages (CD68^+^/CD80^+^) (Supplementary Fig. 3B).

### Antitumor effects of BETi on ovarian tumor growth in an orthotopic in vivo model

**Fig. 4:**
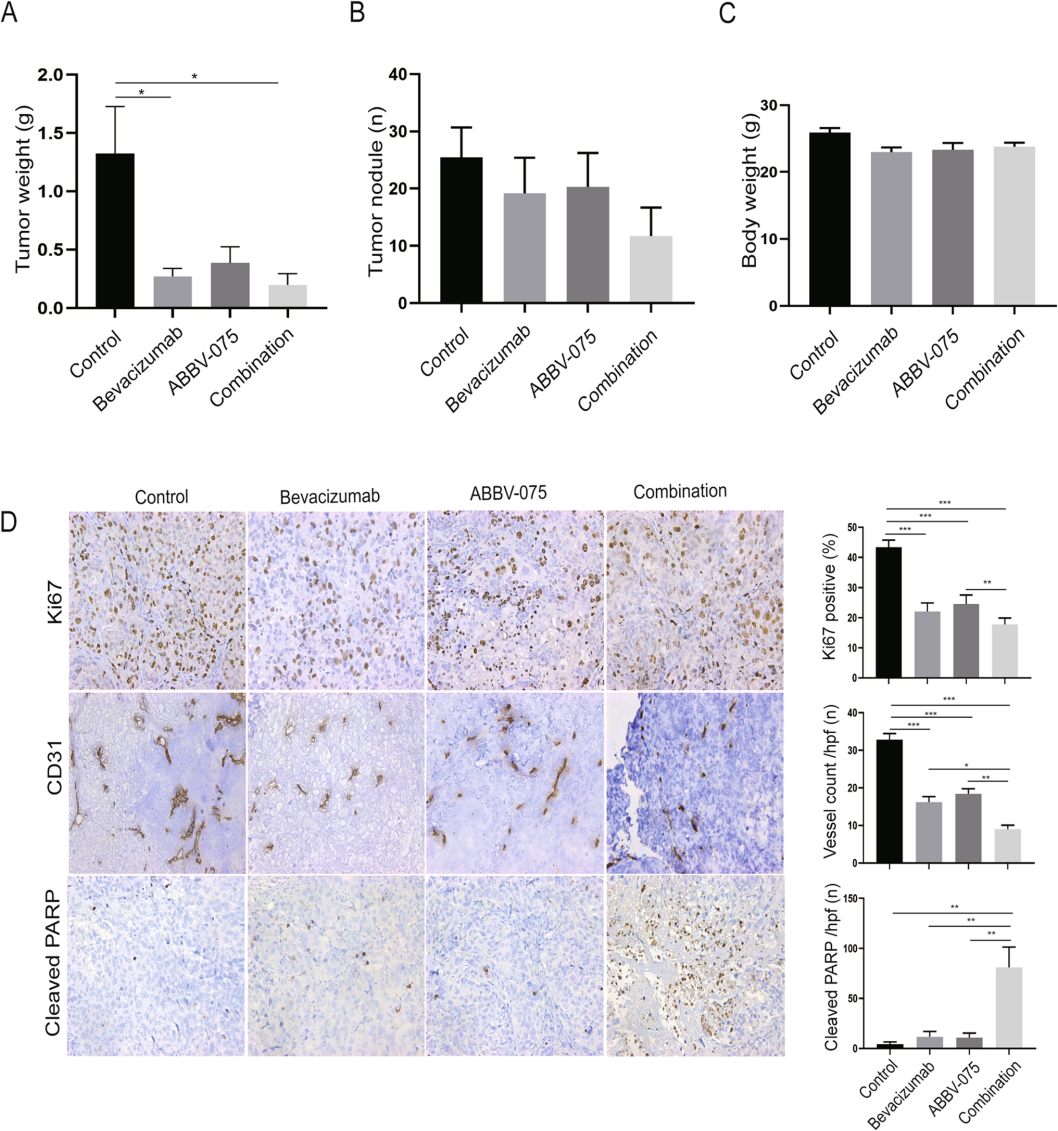
Effects of BETi on ovarian tumor growth in vivo ovarian cancer model. **A** Effect of ABBV-075 and AVA therapy on tumor growth in mice bearing SKOV3ip1 tumors. Control vehicle (*n* = 10), bevacizumab alone (n = 9), ABBV-075 (*n* = 8), and combination (*n* = 7) groups. **B** Graphs of tumor nodule counts. **C** Graphs of mouse body weights. **D** Representative images and quantification graphs of IHC staining for cell proliferation marker (Ki67), vessel density (CD31), and apoptotic marker (cleaved PARP) in the tumor tissue (× 200 magnification). Bar graphs: mean ± SEM. *p < 0.05, **p < 0.01, and ***p < 0.001

We next sought to examine the antitumor effects of BETi (ABBV-075) and AVA (bevacizumab) alone or in combination on the growth of human ovarian tumors in vivo. First, we established the SKOV3ip1 intraperitoneal ovarian tumor model in female athymic nude mice for co-treatment of ABBV-075 and bevacizumab. We allowed the implanted tumors to grow for one week after tumor cell inoculation. Then, mice were randomized into four groups: vehicle control, AVA alone, BETi alone, and combination therapy. Tumor-bearing mice were treated with ABBV-075 1 mg/kg alone (oral gavage, every other day), bevacizumab 6.25 mg/kg alone (intraperitoneal injection, twice per week), or combination therapy for six weeks. Compared with the vehicle control group, mice that received ABBV-075 treatment had a 70.9% decrease in tumor weights and a 20.3% decrease in tumor nodules; mice that received AVA treatment had a 79.5% decrease in tumor weights (*p* < 0.05) and a 19.1% decrease in tumor nodules; mice that received a combination of ABBV-075 and AVA had the most significant (85.1%) decrease in tumor weights (*p* < 0.05) (Fig. 4A) and a 54.1% decrease in tumor nodules (*p* > 0.05) (Fig. 4B). The body weights of the mice had no significant differences between groups during treatment or at the end of treatment (Fig. 4C, Supplementary Fig. 4A).

To further investigate the biological effects of BETi and AVA in the SKOV3ip1 tumor model, we performed IHC staining on paraffin sections of dissected tumors for markers of cell proliferation (Ki67), apoptosis (cleaved PARP), and vessel density (CD31). Compared with the control group, significantly decreased expression in proliferation and angiogenesis markers was noted with ABBV-075, AVA, or the combination treatment (*p* < 0.001) (Fig. 4D). In addition, compared with ABBV-075 alone, the combination of ABBV-075 and AVA significantly decreased proliferation (*p* < 0.01) (Fig. 4D). Compared with AVA alone or ABBV-075 alone, the combination of ABBV-075 and AVA significantly decreased angiogenesis (*p* < 0.05) (Fig. 4D). Consistent with the in vitro apoptosis assay, we found that cleaved PARP was significantly increased in ovarian tumors after treatment with the combination of ABBV-075 and AVA, compared with the control or monotherapy groups (*p* < 0.01) (Fig. 4D).

### BETi overcomes resistance to AVA therapy and prolongs survival in adaptive resistance model of ovarian cancer

**Fig. 5:**
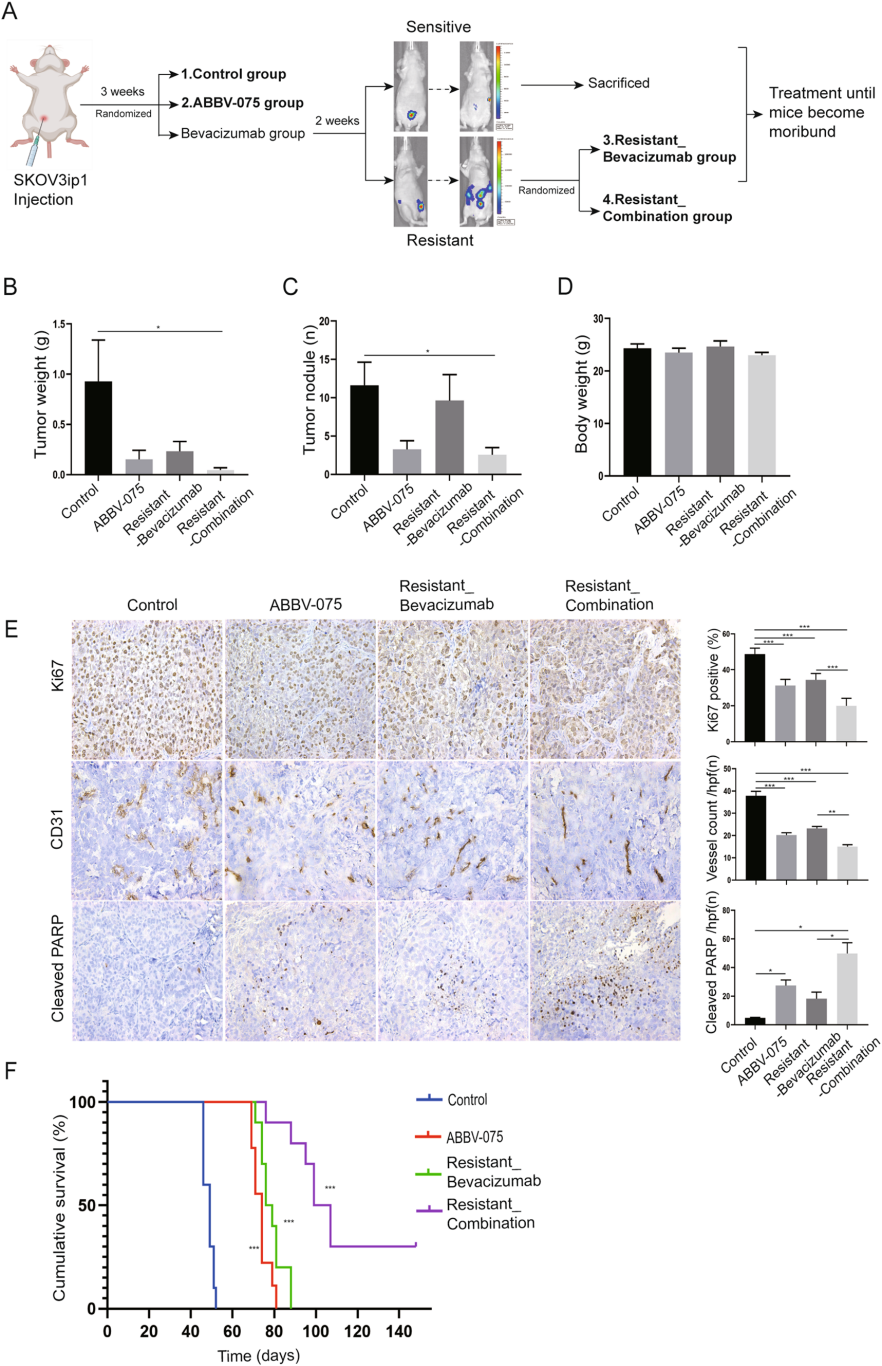
BETi overcomes resistance to AVA therapy and prolongs survival in adaptive resistance model of ovarian cancer. **A** Schematic outline for the resistance model. **B** Effect of ABBV-075 and AVA therapy on ovarian tumor growth in the setting of adaptive resistance to AVA therapy. Control vehicle (*n* = 8), ABBV-075 (*n* = 8), resistant-bevacizumab alone (*n* = 8), and resistant-combination treatment (*n* = 9). Graphs of tumor weights. **C** Graphs of tumor nodule counts. **D** Graphs of mouse body weights. **E** Representative images and quantification graphs of IHC staining for cell proliferation marker (Ki67), vessel density (CD31), and apoptotic marker (cleaved PARP) in the tumor tissue of the resistance model (× 200 magnification). **F** Kaplan–Meier survival analysis of mice receiving control vehicle (*n* = 10), ABBV-075 alone (*n* = 9), resistant-bevacizumab alone (*n* = 10), and resistant-combination treatment (*n* = 10) in adaptive resistance ovarian cancer survival model. **G** IHC stain analysis of macrophage infiltration (F4/80) and M2-like macrophages (ARG1) in tumor tissues from mice with different survival in the adaptive resistance ovarian cancer model (× 200 magnification). Bar graphs: mean ± SEM. *p < 0.05, **p < 0.01, and ***p < 0.001

To investigate whether ABBV-075 treatment can overcome resistance to AVA therapy, we established an adaptive resistance model. Mice were injected with SKOV3ip1 ovarian cancer cells intraperitoneally. Three weeks after cell injection, IVIS imaging confirmed the xenograft tumor; mice were then randomized to the following groups: vehicle control, BETi (ABBV-075 1 mg/kg, oral gavage, daily), and AVA (bevacizumab 6.25 mg/kg, intraperitoneal injection, twice per week) monotherapy groups. The mice were monitored by IVIS imaging weekly to determine response to treatment according to the bioluminescence signal. After two weeks of treatment, mice in the AVA group were separated into sensitive and resistant groups. We determined mice to be sensitive to treatment when the bioluminescence signal was decreased or stable; mice that were resistant to treatment had an increase in bioluminescence signal. Sensitive mice were euthanized after they were separated, whereas resistant mice were randomized to continue to receive AVA alone (resistant-bevacizumab group) or in addition to BETi therapy (resistant-combination group). Mice received treatment until they became moribund (Fig. 5A).

In the adaptive resistance model, mice that received combination treatment had a 95.1% significant lower tumor weight and 78.0% lower tumor nodules compared with the vehicle control group (*p* < 0.05); mice that received ABBV-075 alone had an 83.6% and 72.0% decrease in tumor weight and tumor nodules (*p* > 0.05), respectively; mice that received AVA alone had a 75.1% and 17.2% decrease (*p* > 0.05), respectively (Fig. 5B, C). The body weights of mice were not significantly different between groups (Fig. 5D, Supplementary Fig. 4B). Tumors treated with ABBV-075 and with AVA had lower Ki67 and CD31 compared with levels observed in the vehicle control treatment group (*p* < 0.001); combination treatment resulted in the most significant decreases in these markers (*p* < 0.001) (Fig. 5E). Furthermore, compared with vehicle control or AVA alone in the resistant mice, combination therapy in the resistant mice showed significant increases in the apoptosis marker (cleaved PARP, *p* < 0.05), whereas AVA alone in the resistant mice resulted in no significant increase in apoptosis (Fig. 5E).

Furthermore, to investigate the long-term therapeutic effects of ABBV-075 in the ovarian tumor model, we set up an adaptive resistance survival model. Kaplan–Meier curves were used to analyze the survival difference between vehicle control and treatment groups. In the adaptive resistance survival model, mice that received ABBV-075 in addition to AVA at the emergence of resistance had significantly longer survival than did mice that received therapy with the vehicle control, ABBV-075 alone, or continued AVA alone (*p* < 0.001) (Fig. 5F). Body weights of the mice had no significant decrease during treatment (Supplementary Fig. 4C).

### ABBV-075 selectively targeted macrophage infiltration-related cytokines/ chemokines in ovarian cancer

**Fig. 6:**
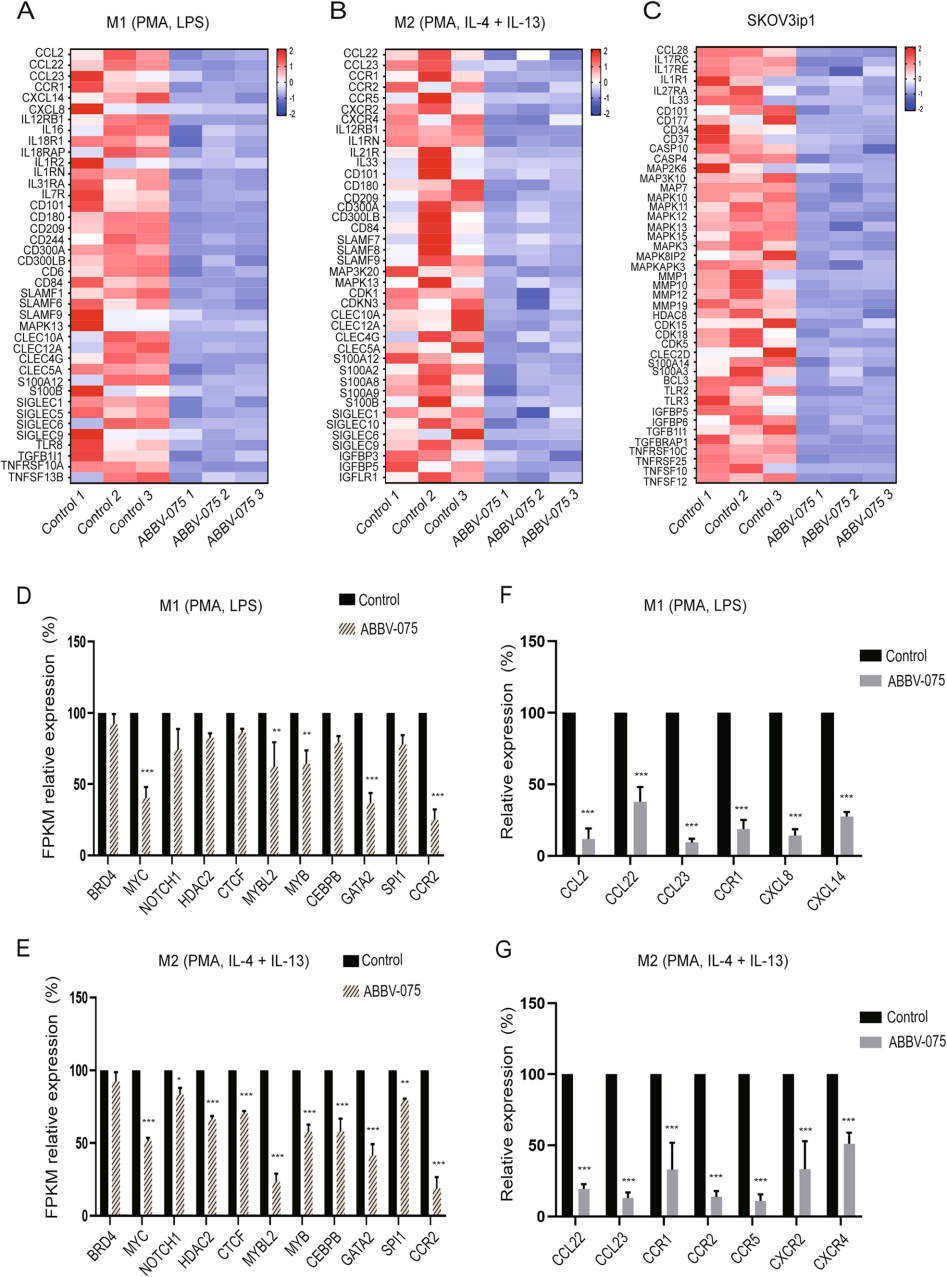
Effects of BETi on inflammatory, immune, and downstream regulators in macrophages and ovarian cancer cells by RNA-seq analysis. **A** Heatmap of differentially expressed genes related to inflammatory and immune regulation in M1 macrophages (PMA, LPS). Control vs. ABBV-075 (0.01 µM), 24 h. **B** Heatmap of differentially expressed genes related to inflammatory and immune regulation in M2 macrophages (PMA, IL-4 + IL-13). Control vs. ABBV-075 (0.01 µM), 24 h. **C** Heatmap of differentially expressed genes related to inflammatory, immune regulation, and downstream regulation in SKOV3ip1 ovarian cancer cells. Control vs. ABBV-075 (1.0 µM), 24 h. The heatmap data are displayed as z-score values. Differentially expressed genes are selected on the basis of adjusted *p* < 0.05 and Log2 fold change ≥ 1.5. **D, E** Transcription factors in M1 (PMA, LPS) and M2 (PMA, IL-4 + IL-13) macrophages after ABBV-075 (0.01 µM) treatment for 24 h calculated by FPKM value of RNA-seq. **F, G** Validation of chemokine/receptor expression in M1 (PMA, LPS) and M2 (PMA, IL-4 + IL-13) macrophages after ABBV-075 (0.01 µM) treatment for 24 h by qRT-PCR analysis. Bar graphs: mean ± SEM. *p < 0.05, **p < 0.01, and ***p < 0.001

To identify potential mechanisms by which ABBV-075 selectively targeted macrophages and ovarian cancer cells, we evaluated the effects of ABBV-075 (24 h treatment) on gene expression by RNA sequencing. There were 343 downregulated genes and 89 upregulated genes in M1-like macrophages, and 492 downregulated genes and 117 upregulated genes in M2-like macrophages; 974 downregulated genes and 302 upregulated genes were identified in ovarian cancer cells (adjusted *p* < 0.05 and Log2 fold change ≥ 1.5). Interestingly, we found that inflammatory cytokines and chemokines related to macrophage infiltration and activation were downregulated by ABBV-075 in macrophages (adjusted *p* < 0.05 and Log2 fold change ≥ 1.5) (Fig. 6A, B), which included immune regulation and inflammatory-related cytokines or chemokines, SLAMF family receptors, interleukin and its receptor family, C-type lectin-like domain-containing family, S100 family genes, and sialic acid-binding immunoglobulin-type lectins (Siglec) family. The mitogen-activated protein kinase (MAPK) family, matrix metalloproteinase (MMP) family, cyclin-dependent kinases and histone deacetylase 8 were significantly downregulated by ABBV-075 in SKOV3ip1 cells (adjusted *p* < 0.05 and log2 fold change ≥ 1.5) (Fig. 6C). The genes in the heatmaps were selected on the basis of their biological and molecular function related to immune regulation, inflammatory regulation, and tumor oncogenic regulation.

As expected, there was significantly decreased expression of CCR2 in macrophages according to gene expression level (FPKM) in RNA-seq analysis (*p* < 0.001) (Fig. 6D, E), consistent with results from qRT-PCR, flow cytometry, and Western blot analysis. In addition, the CCR2-related transcription factors were significantly decreased in macrophages (*p* < 0.05) (Fig. 6D, E). Furthermore, we validated that the expression of chemokines was significantly decreased in macrophages after ABBV-075 treatment by qRT-PCR (*p* < 0.001) (Fig. 6F, G). Potential biological functions for these chemokines in macrophages are shown in Supplementary table 2. These findings suggest that ABBV-075 targeted mostly immune-related genes in macrophages and the MAPK family in cancer cells.

### Macrophage infiltration correlates with clinical outcome in ovarian cancer

**Fig. 7:**
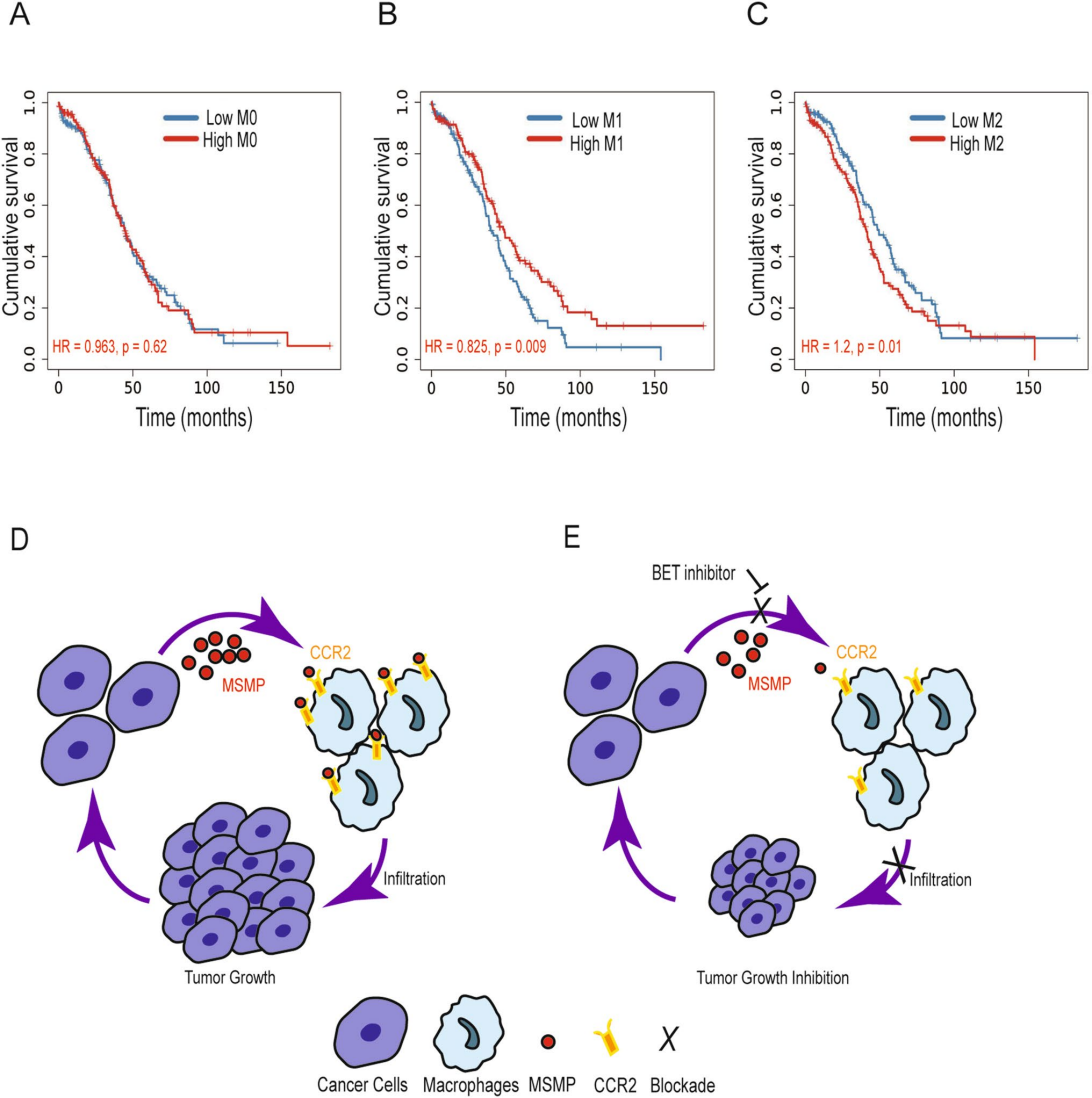
Macrophage infiltration in patients with ovarian cancer. **A** Kaplan–Meier survival analysis of patients stratified by the infiltration level of M0 macrophages in ovarian cancer from the TCGA cohort (*n* = 303) using the CIBERSORT analytical algorithm by TIMER2.0. **B** Kaplan–Meier survival analysis of patients stratified by the infiltration level of M1 macrophages in ovarian cancer from the TCGA cohort (*n* = 303) using the CIBERSORT analytical algorithm by TIMER2.0. **C** Kaplan–Meier survival analysis of patients stratified by the infiltration level of M2 macrophages in ovarian cancer from the TCGA cohort (*n* = 303) using the CIBERSORT analytical algorithm by TIMER2.0. **D** A proposed model for the role of MSMP/CCR2 signaling in macrophages and cancer cells. **E** BETi suppresses MSMP/CCR2 signaling in macrophages and cancer cells

To evaluate the clinical significance of macrophages on survival rate in patients with ovarian cancer, we used the TIMER2.0 analytical tool (Li et al. 2020). The clinical relevance of macrophage infiltration was explored in the TCGA cohort by a multivariable Cox proportional hazard model. Clinical factors (age and stage) were included as covariates. Kaplan–Meier curves, hazard ratio (HR), and *p* value of overall survival was calculated on the basis of macrophage infiltration level (low level vs. high level) with use of the CIBERSORT algorithm (Chen et al. 2018; Newman et al. 2015; Sturm et al. 2019). The survival analysis revealed that M0 macrophage infiltration in ovarian cancer had no significant difference in overall survival (*p* =0.62) (Fig. 7A), whereas high levels of M1-like macrophages were correlated with a significant increase in overall survival (*p* =0.009) (Fig. 7B). Conversely, high levels of M2-like macrophages were correlated with significantly lower overall survival (*p* =0.01) (Fig. 7C), which was consistent with a recent report in ovarian cancer (Maccio et al. 2020). Collectively, these findings further support the translational relevance of our preclinical findings indicating improving anti-angiogenesis therapy by targeting macrophages in adaptive resistance ovarian cancer models (Fig. 7D, E).

## Discussion

In this study, we identified a previously unrecognized role of BETi in overcoming adaptive resistance to AVA therapy in ovarian cancer by selectively targeting CCR2^+^ macrophages. BETi have been developed as a novel class of potential anticancer agents and have shown robust preclinical antitumor activity (Shorstova et al. 2021). Many clinical trials of BETi are ongoing (Stathis and Bertoni 2018). However, most previous studies of BETi mainly examined BRD2/BRD3/BRD4 proteins in cancer cells; few studies have considered the effects of BETi on macrophages. We found that the combination of ABBV-075 with AVA decreased the infiltration of macrophages, and improved efficacy of AVA therapy in ovarian cancer models; the BETi treatment also induced macrophage reprogramming toward M1-like macrophages and inhibited M2-like macrophage infiltration.

It is well known that ovarian cancer is an immunologically unresponsive “cold” tumor that is infiltrated with immunosuppressive cells, such as TAMs, and lacks activated cytotoxic lymphocytes (Ghisoni et al. 2019). Growing evidence suggests that macrophage infiltration in ovarian cancer could facilitate resistance to AVA therapy and promote tumor progression (Chambers et al. 1997; Dalton et al. 2017; Yin et al. 2016); therefore, it is crucial to target macrophages to overcome resistance to AVA therapy. In this study, we demonstrated that the combination of ABBV-075 with AVA decreased the infiltration of macrophages in ovarian tumors. Importantly, we determined that ABBV-075 induced macrophage reprogramming toward the M1-like phenotype and that high M1 macrophage count was associated with longer patient survival; in contrast, high M2 macrophage count was associated with shorter survival of patients with ovarian cancer. Consistent with a recent study, a high prevalence of M1 cells and a high M1/M2 ratio was associated with better prognosis in ovarian cancer patients; furthermore, platinum-sensitive tumors had a higher M1/M2 ratio than did platinum-resistant tumors (Maccio et al. 2020). Moreover, other studies have reported that reprogramming of macrophages in the tumor microenvironment could be an effective strategy for supporting a cytotoxic T-cell response and antitumor activity (Sun et al. 2021), which complements the depletion of TAMs (Ardighieri et al. 2021; Jaynes et al. 2020; Wanderley et al. 2018; Zhu et al. 2014). Further studies are warranted to investigate the effects of ABBV-075 on comprehensive immune regulation, especially cytotoxic T-cell response.

Previous studies, which primarily targeted the colony stimulating factor 1 receptor (CSF1R)/colony stimulating factor 1 (CSF1) axis with a CSF1R inhibitor, suggested that targeting the CSF1R/CSF1 axis could be a promising approach (Pyonteck et al. 2013; Ries et al. 2014; Yan et al. 2017). However, dose escalation of CSF1R inhibitor has been limited because of possible toxicity from depletion of all macrophages (Cassetta and Pollard 2018). Moreover, CSF1R-specific inhibitors can induce the rebound of CCR2-enriched classical monocytes (Ries et al. 2014). Novel therapeutic strategy targeting the MSMP/CCR2 axis and CCR2^+^ monocytes/macrophages with BETi may avoid the rebound effects of CSF1R inhibitor. The signaling of CCL2/CCR2 is well known to promote macrophage infiltration and activation, consequently contributing to tumor growth and progression (Li et al. 2017; Petty and Yang 2017; She et al. 2020; Yang et al. 2020). In this study, we demonstrated that BETi not only reduced CCR2 expression in macrophages, but also affected immune regulation and inflammation-related cytokines and chemokines, which may crosstalk with CCR2. ABBV-075 inhibited the infiltration of macrophages and their interaction with ovarian cancer cells through multiple mechanisms, as demonstrated by our results (Mitamura et al. 2018). Therefore, CCR2^+^ macrophages and CCR2-interacting ligands could serve as potential predictors of emergence of resistance to AVA therapy.

In summary, our study suggests a previously unrecognized role for BETi (ABBV-075) in enhancing the efficacy of AVA therapy in ovarian cancer models. Further study is warranted to evaluate the clinical utility of the combination of ABBV-075 and AVA.

## Supplementary Information

Below is the link to the electronic supplementary material.

Supplementary file1 (DOCX 3504 kb)

Supplementary file2 (TIF 26961 kb)

Supplementary file3 (TIF 33522 kb)

Supplementary file4 (TIF 18253 kb)

Supplementary file5 (TIF 14688 kb)

## Abbreviations

VEGF: Vascular endothelial growth factor
FDA: The U.S. Food and Drug Administration
AVA: Anti-VEGF antibody
TAM: Tumor-associated macrophages
CCL2: C–C motif chemokine ligand 2
CCR2: C–C motif chemokine receptor 2
MSMP: Microseminoprotein
BETi: Bromodomain and extra-terminal domain (BET) inhibitors
ARG1: Arginase-1
PMA: Phorbol 12-myristate 13-acetate
LPS: Lipopolysaccharides
IL-4: Interleukin 4
IL-13: Interleukin 13
IHC: Immunohistochemical
IVIS: In vivo imaging system
FPKM: The fragments per kilobase of transcript sequence per million
DICE: Database of Immune Cell Expression
TIMER2.0: Tumor Immune Estimation Resource 2.0
TCGA: The Cancer Genome Atlas
CSF1R: Colony stimulating factor 1 (CSF1) receptor
