# Supplementary material for: Targeting CCR2+ macrophages with BET inhibitor overcomes adaptive resistance to anti-VEGF therapy in ovarian cancer
Source: J Cancer Res Clin Oncol. 2022 Jan 30;148(4):803–21. doi: 10.1007/s00432-021-03885-z (PMC8930900; doi:10.1007/s00432-021-03885-z)
Supplement: Supplementary file 1 — Supplementary file1 (DOCX 3504 kb) [file 432_2021_3885_MOESM1_ESM.docx]

**Targeting CCR2^+^ macrophages with BET inhibitor overcomes adaptive resistance to anti-VEGF therapy in ovarian cancer**

Yutuan Wu^1,2,3^, Nicholas B. Jennings^1^, Yunjie Sun^1^, Santosh K. Dasari^1^, Emine Bayraktar^1^, Sara Corvigno^1^, Elaine Stur^1^, Deanna Glassman^1^, Lingegowda S. Mangala^1^, Adrian Lankenau Ahumada^1^, Shannon N. Westin^1^, Anil K. Sood^1,4,5^, Wei Hu^1^

^1^Department of Gynecologic Oncology and Reproductive Medicine, The University of Texas MD Anderson Cancer Center, Houston, TX, USA. ^2^Department of Gynecologic Oncology, Fudan University Shanghai Cancer Center, Shanghai, People’s Republic of China. ^3^Department of Oncology, Shanghai Medical College, Fudan University, Shanghai, People’s Republic of China. ^4^Department of Cancer Biology and ^5^Center for RNA Interference and Non-Coding RNA, The University of Texas MD Anderson Cancer Center, Houston, TX, USA.

**Corresponding authors:** Anil K. Sood and Wei Hu. Department of Gynecologic Oncology and Reproductive Medicine, The University of Texas MD Anderson Cancer Center, 1155 Herman Pressler Boulevard, Unit 1362, Houston, TX 77030. Phone: 713-563-8365; Fax: 713-792-7586; E-mail: [asood@mdanderson.org](mailto:asood@mdanderson.org) or [weihu@mdanderson.org](mailto:weihu@mdanderson.org)

**Running title:** Targeting CCR2^+^ macrophages with BET inhibitor in ovarian cancer


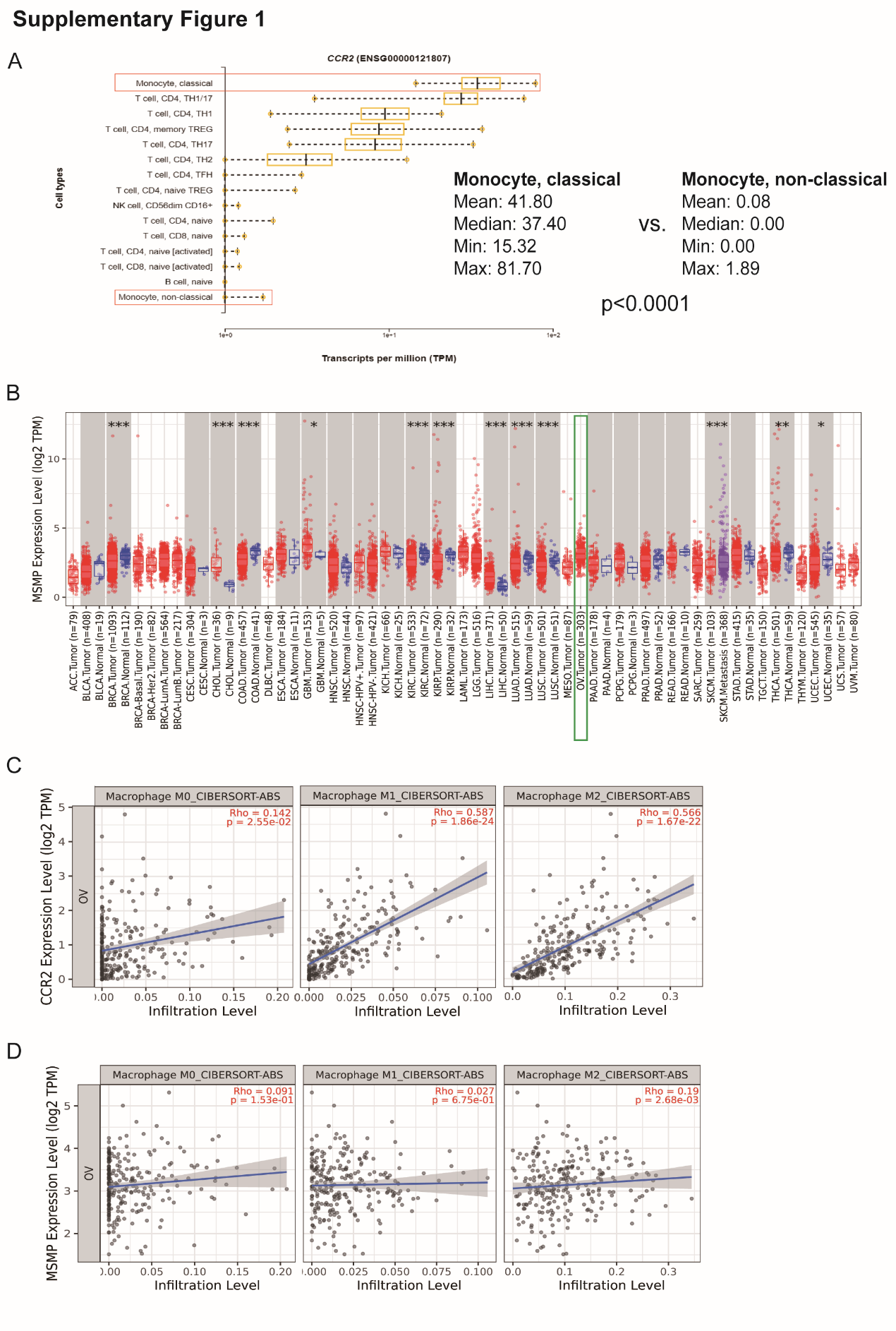
**Supplementary Figure 1.** CCR2 and MSMP expression and macrophage infiltration in ovarian cancer. **A** Expression of CCR2 in classical monocytes and non-classical monocytes (red box) by DICE analysis. **B** Expression of MSMP in ovarian cancer (green box) from the TCGA cohort. **C** Expression of CCR2 and its correlation with macrophage infiltration in ovarian cancer from the TCGA cohort using CIBERSORT-ABS analytical algorithm by TIMER2.0. **D** Expression of MSMP and its correlation with macrophage infiltration in ovarian cancer from the TCGA cohort using CIBERSORT-ABS analytical algorithm by TIMER2.0.


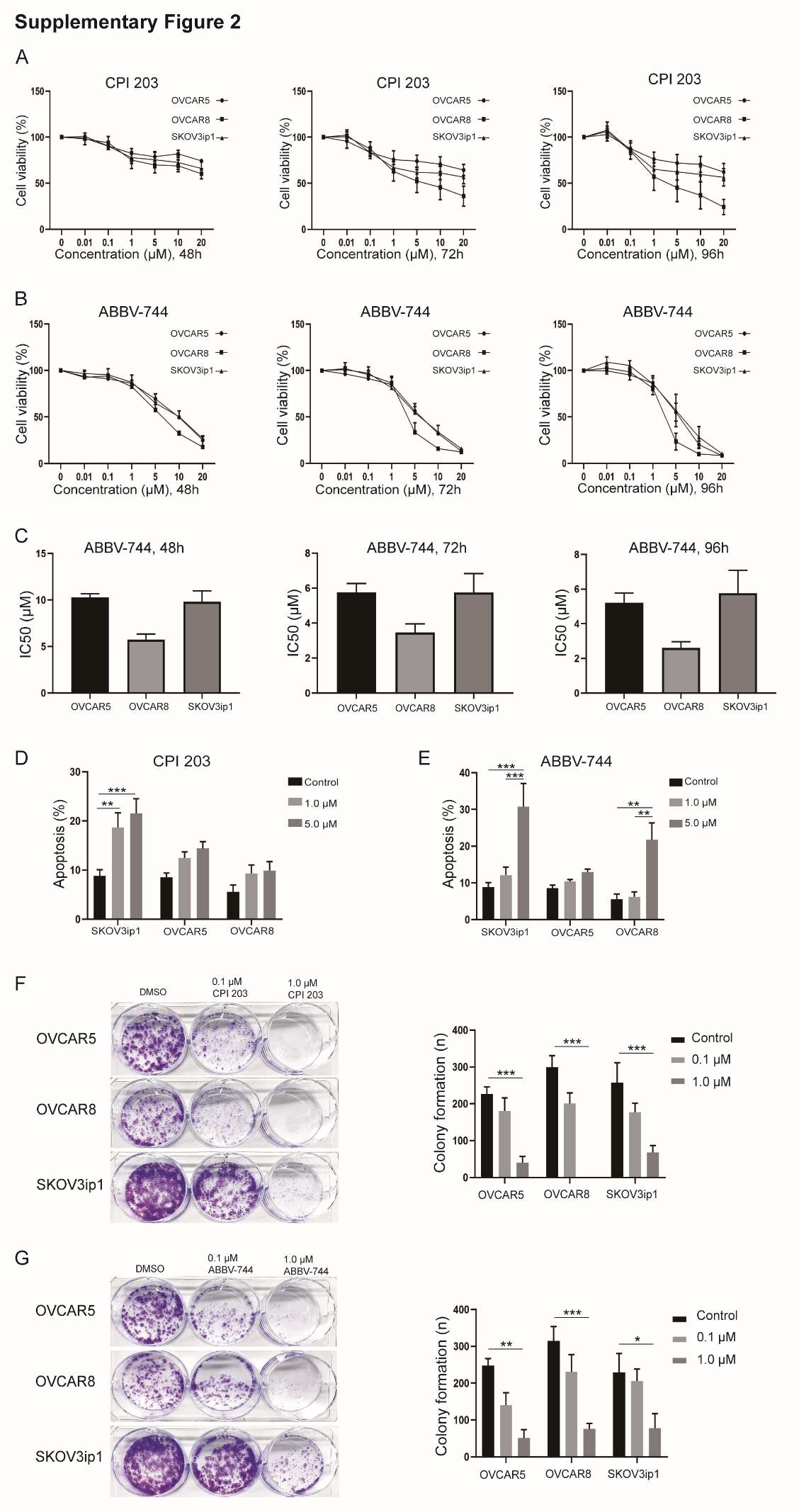
**Supplementary Figure 2.** Antitumor effects of BETi on ovarian cancer cells. **A** MTT cell viability assay of SKOV3ip1, OVCAR5, and OVCAR8 cells after treatment with CPI 203 for 48, 72, and 96 h. **B** MTT cell viability assay of SKOV3ip1, OVCAR5, and OVCAR8 cells after treatment with ABBV-744 for 48, 72, and 96 h. **C** IC50 values of ABBV-744 for 48, 72, and 96 h of treatment in SKOV3ip1, OVCAR5, and OVCAR8 cells. **D** Flow cytometry analysis of apoptosis in SKOV3ip1, OVCAR5, and OVCAR8 cells after treatment with CPI 203 for 48 h. **E** Flow cytometry analysis of apoptosis in SKOV3ip1, OVCAR5, and OVCAR8 cells after treatment with ABBV-744 for 48 h. **F** Effect of 7-10 d of CPI 203 treatment on colony formation in SKOV3ip1, OVCAR5, and OVCAR8 cells. **G** Effect of 7-10 d of ABBV-744 treatment on colony formation in SKOV3ip1, OVCAR5, and OVCAR8 cells. Experiments were performed in triplicate by independent assays. Bar graphs: mean ± SEM. **p* <0.05, ** *p* <0.01, and *** *p* <0.001.


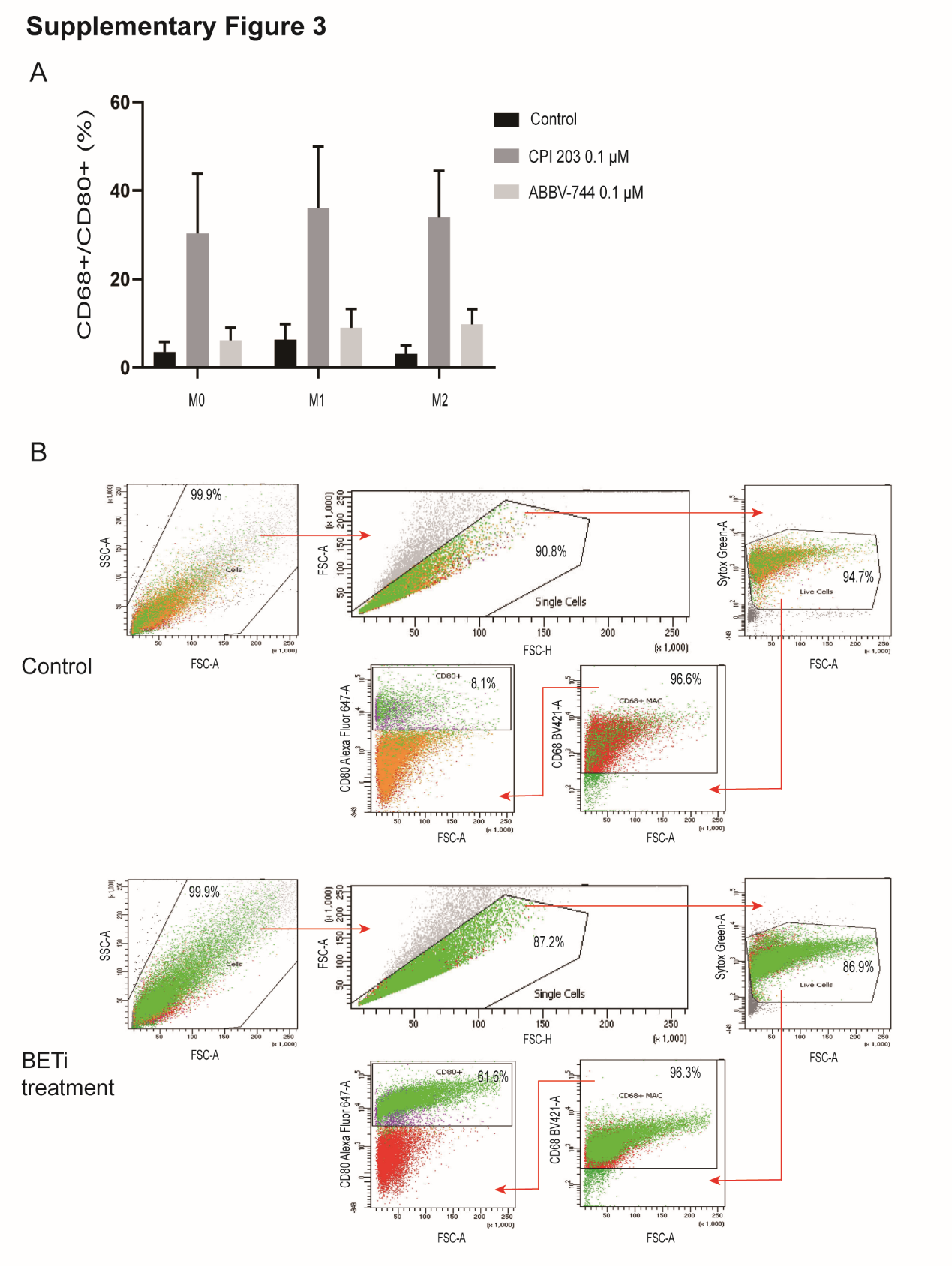
**Supplementary Figure 3.** Effects of BETi on macrophages. **A** Flow cytometry analysis of CD68+/CD80+ M1-like macrophage proportion after treatment with 0.1 µM CPI 203 or ABBV-744 for 48 h. Bar graphs: mean ± SEM. **B** Gating strategy for flow cytometry analysis of CD68^+^/CD80^+^ M1-like macrophage proportion after vehicle control, and BETi (ABBV-075) treatment. Experiments were performed in triplicate by independent assays.


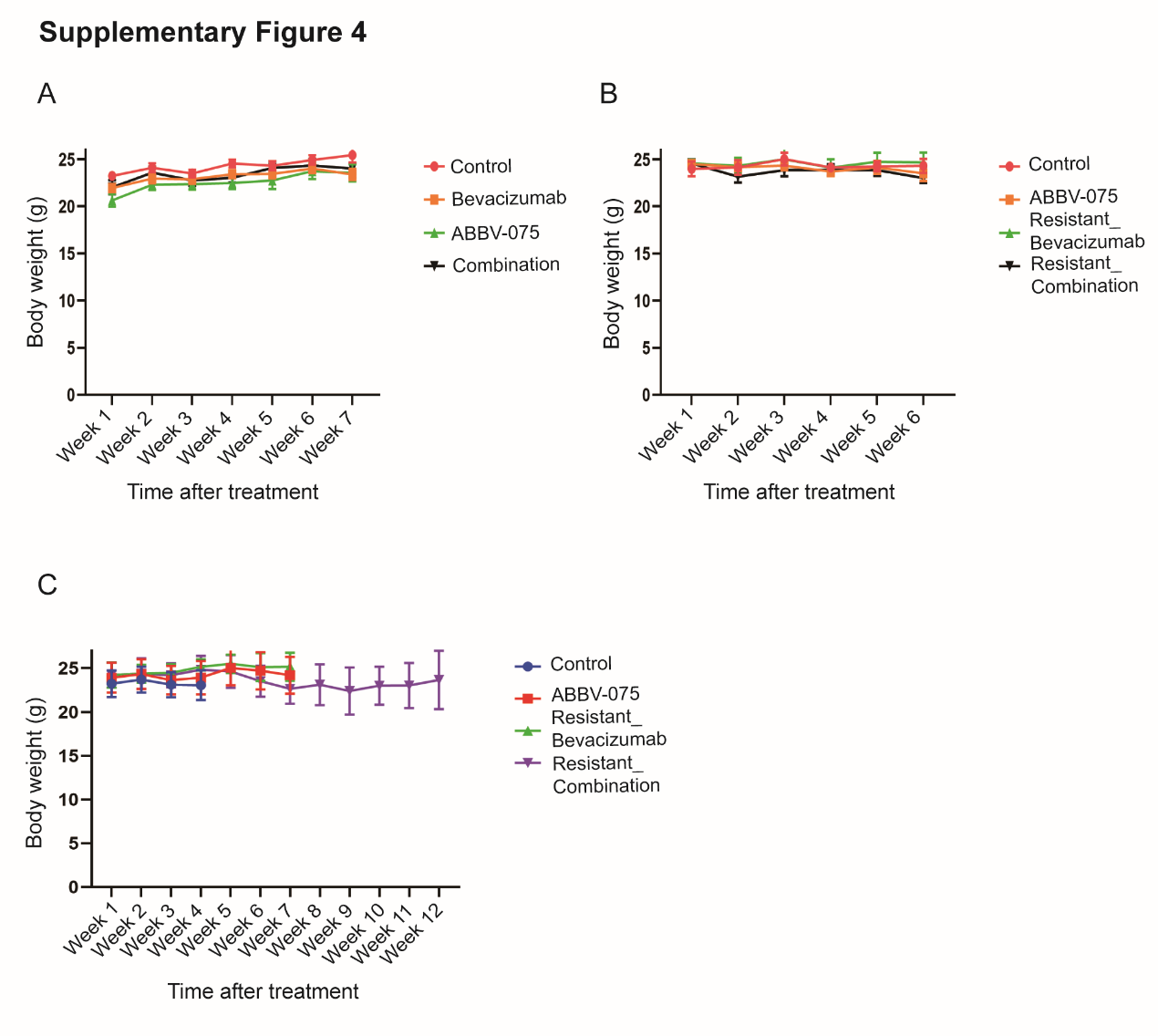
**Supplementary Figure 4.** Effects of BETi on body weight *in vivo*. **A** Body weight of mice in the xenograft co-treatment model. Control vehicle (n=10), bevacizumab alone (n=9), ABBV-075 (n=8), and combination (n=7) groups. Data shown as mean and SD. **B** Body weight of mice in adaptive resistance co-treatment model. Control vehicle (n=8), ABBV-075 (n=8), resistant-bevacizumab alone (n=8), and resistant-combination treatment (n=9). Data shown as mean and SD. **C** Body weight of mice in adaptive resistance survival model. Control vehicle (n=10), ABBV-075 (n=9), resistant-bevacizumab alone (n=10), and resistant-combination treatment (n=10). Data shown as mean ± SD.

**Supplementary Table 1.** Primers for qRT-PCR.

| **Primer Name** | **5' Mod to 3' Mod** |
| --- | --- |
| hCCL2-F | CAGCCAGATGCAATCAATGCC |
| hCCL2-R | TGGAATCCTGAACCCACTTCT |
|  |  |
| hCCL22-F | ATCGCCTACAGACTGCACTC |
| hCCL22-R | GACGGTAACGGACGTAATCAC |
|  |  |
| hCCL23-F | CATCTCCTACACCCCACGAAG |
| hCCL23-R | GGGTTGGCACAGAAACGTC |
|  |  |
| hCCR1-F | GACTATGACACGACCACAGAGT |
| hCCR1-R | CCAACCAGGCCAATGACAAATA |
|  |  |
| hCCR5-F | GTTGGACCAAGCTATGCAGGT |
| hCCR5-R | GCAGAAGCGTTTGGCAATGT |
|  |  |
| hCXCL8-F | TTTTGCCAAGGAGTGCTAAAGA |
| hCXCL8-R | AACCCTCTGCACCCAGTTTTC |
|  |  |
| hCXCL14-F | CGCTACAGCGACGTGAAGAA |
| hCXCL14-R | GTTCCAGGCGTTGTACCAC |
|  |  |
| hCXCR2-F | CCTGTCTTACTTTTCCGAAGGAC |
| hCXCR2-R | TTGCTGTATTGTTGCCCATGT |
|  |  |
| hCXCR4-F | ACTACACCGAGGAAATGGGCT |
| hCXCR4-R | CCCACAATGCCAGTTAAGAAGA |
|  |  |
| h18S-F | CGCCGCTAGAGGTGAAATTC |
| h18S-R | TTGGCAAATGCTTTCGCTC |
|  |  |
| hCCR2-F | ACCGAGAACGAGATGTGGAC |
| hCCR2-R | CTGAGACAAGCCACAAGCTG |
|  |  |
| hMSMP-F | CTTCCAAGCTCAAGCCCCCT |
| hMSMP-R | ATGCAGACAGGTGCAATGGA |
|  |  |
| hCD80-F | GGGGAAATGTCGCCTCTCTG |
| hCD80-R | GTGGATTTAGTTTCACAGCTTGC |
|  |  |
| hCD86-F | TCATTGCCGAGGAAGGCTTG |
| hCD86-R | TCAGGTTGACTGAAGTTAGCAGA |
|  |  |
| hCD206-F | TTCCTTTGGACGGATGGACG |
| hCD206-R | GTCAAGGAAGGGTCGGATCG |
|  |  |
| hCD163-F | GAAGACAGAGACAGCGGCTT |
| hCD163-R | AGGTATCTTAAAGGCTCACTGG |

**Supplementary Table 2.** Potential functions for the downregulated chemokines/ receptors in macrophages

| **Marker** | **Function** | **Reference** |
| --- | --- | --- |
| CCL2 | Promoted recruitment of monocytes/macrophages | (Gregory et al., 2006; H. Yang et al., 2020) |
| CCL22 | Mainly synthesized by M2-like macrophage and induced migration of regulatory T cells | (Ishida et al., 2006; Kimura et al., 2019) |
| CCL23 | Interacted with CCR1 and promoted cancer cells migration | (Krishnan et al., 2020; Xuan et al., 2015) |
| CCR1 | Interacted with chemokine ligands and promoted tumor progression | (Krishnan et al., 2020; Xuan et al., 2015) |
| CCR2 | Interacted with CCL2, MSMP and promoted infiltration of monocytes/macrophages | (Boniakowski et al., 2018; Hui Yang et al., 2020) |
| CCR5 | Closely related with M2-like macrophage and recruitment of macrophages | (Korbecki et al., 2020; Xuan et al., 2015) |
| CXCL8 | Secreted by tumor-associated macrophages and induced immunosuppressive microenvironment | (Lin et al., 2019; Zhang et al., 2020) |
| CXCL14 | Stimulation of monocyte migration and tumor growth. | (Augsten et al., 2009; Lu et al., 2016) |
| CXCR2 | Strongly related with tumor-associated macrophage infiltration and supported tumorigenesis | (Di Mitri et al., 2019; Zhang et al., 2020) |
| CXCR4 | Closely related with M2-like macrophages and promoted angiogenesis | (Owen et al., 2013; Xuan et al., 2015) |

**References**

Augsten, M., Hägglöf, C., Olsson, E., Stolz, C., Tsagozis, P., Levchenko, T., Frederick, M. J., Borg, A., Micke, P., Egevad, L., & Ostman, A. (2009). CXCL14 is an autocrine growth factor for fibroblasts and acts as a multi-modal stimulator of prostate tumor growth. *Proc Natl Acad Sci U S A, 106*(9), 3414-3419. doi:10.1073/pnas.0813144106

Boniakowski, A. E., Kimball, A. S., Joshi, A., Schaller, M., Davis, F. M., denDekker, A., Obi, A. T., Moore, B. B., Kunkel, S. L., & Gallagher, K. A. (2018). Murine macrophage chemokine receptor CCR2 plays a crucial role in macrophage recruitment and regulated inflammation in wound healing. *Eur J Immunol, 48*(9), 1445-1455. doi:10.1002/eji.201747400

Di Mitri, D., Mirenda, M., Vasilevska, J., Calcinotto, A., Delaleu, N., Revandkar, A., Gil, V., Boysen, G., Losa, M., Mosole, S., Pasquini, E., D'Antuono, R., Masetti, M., Zagato, E., Chiorino, G., Ostano, P., Rinaldi, A., Gnetti, L., Graupera, M., Martins Figueiredo Fonseca, A. R., Pereira Mestre, R., Waugh, D., Barry, S., De Bono, J., & Alimonti, A. (2019). Re-education of Tumor-Associated Macrophages by CXCR2 Blockade Drives Senescence and Tumor Inhibition in Advanced Prostate Cancer. *Cell Rep, 28*(8), 2156-2168.e2155. doi:10.1016/j.celrep.2019.07.068

Gregory, J. L., Morand, E. F., McKeown, S. J., Ralph, J. A., Hall, P., Yang, Y. H., McColl, S. R., & Hickey, M. J. (2006). Macrophage migration inhibitory factor induces macrophage recruitment via CC chemokine ligand 2. *J Immunol, 177*(11), 8072-8079. doi:10.4049/jimmunol.177.11.8072

Ishida, T., & Ueda, R. (2006). CCR4 as a novel molecular target for immunotherapy of cancer. *Cancer Sci, 97*(11), 1139-1146. doi:10.1111/j.1349-7006.2006.00307.x

Kimura, S., Nanbu, U., Noguchi, H., Harada, Y., Kumamoto, K., Sasaguri, Y., & Nakayama, T. (2019). Macrophage CCL22 expression in the tumor microenvironment and implications for survival in patients with squamous cell carcinoma of the tongue. *J Oral Pathol Med, 48*(8), 677-685. doi:10.1111/jop.12885

Korbecki, J., Grochans, S., Gutowska, I., Barczak, K., & Baranowska-Bosiacka, I. (2020). CC Chemokines in a Tumor: A Review of Pro-Cancer and Anti-Cancer Properties of Receptors CCR5, CCR6, CCR7, CCR8, CCR9, and CCR10 Ligands. *Int J Mol Sci, 21*(20). doi:10.3390/ijms21207619

Krishnan, V., Tallapragada, S., Schaar, B., Kamat, K., Chanana, A. M., Zhang, Y., Patel, S., Parkash, V., Rinker-Schaeffer, C., Folkins, A. K., Rankin, E. B., & Dorigo, O. (2020). Omental macrophages secrete chemokine ligands that promote ovarian cancer colonization of the omentum via CCR1. *Commun Biol, 3*(1), 524. doi:10.1038/s42003-020-01246-z

Lin, C., He, H., Liu, H., Li, R., Chen, Y., Qi, Y., Jiang, Q., Chen, L., Zhang, P., Zhang, H., Li, H., Zhang, W., Sun, Y., & Xu, J. (2019). Tumour-associated macrophages-derived CXCL8 determines immune evasion through autonomous PD-L1 expression in gastric cancer. *Gut, 68*(10), 1764-1773. doi:10.1136/gutjnl-2018-316324

Lu, J., Chatterjee, M., Schmid, H., Beck, S., & Gawaz, M. (2016). CXCL14 as an emerging immune and inflammatory modulator. *J Inflamm (Lond), 13*, 1. doi:10.1186/s12950-015-0109-9

Owen, J. L., & Mohamadzadeh, M. (2013). Macrophages and chemokines as mediators of angiogenesis. *Front Physiol, 4*, 159. doi:10.3389/fphys.2013.00159

Xuan, W., Qu, Q., Zheng, B., Xiong, S., & Fan, G. H. (2015). The chemotaxis of M1 and M2 macrophages is regulated by different chemokines. *J Leukoc Biol, 97*(1), 61-69. doi:10.1189/jlb.1A0314-170R

Yang, H., Zhang, Q., Xu, M., Wang, L., Chen, X., Feng, Y., Li, Y., Zhang, X., Cui, W., & Jia, X. (2020). CCL2-CCR2 axis recruits tumor associated macrophages to induce immune evasion through PD-1 signaling in esophageal carcinogenesis. *Mol Cancer, 19*(1), 41. doi:10.1186/s12943-020-01165-x

Yang, H., Zhang, Q., Xu, M., Wang, L., Chen, X., Feng, Y., Li, Y., Zhang, X., Cui, W., & Jia, X. (2020). CCL2-CCR2 axis recruits tumor associated macrophages to induce immune evasion through PD-1 signaling in esophageal carcinogenesis. *Molecular Cancer, 19*(1), 41. doi:10.1186/s12943-020-01165-x

Zhang, M., Huang, L., Ding, G., Huang, H., Cao, G., Sun, X., Lou, N., Wei, Q., Shen, T., Xu, X., Cao, L., & Yan, Q. (2020). Interferon gamma inhibits CXCL8-CXCR2 axis mediated tumor-associated macrophages tumor trafficking and enhances anti-PD1 efficacy in pancreatic cancer. *J Immunother Cancer, 8*(1). doi:10.1136/jitc-2019-000308
