# Supplementary figures and images for: Targeting CCR2+ macrophages with BET inhibitor overcomes adaptive resistance to anti-VEGF therapy in ovarian cancer
Source: J Cancer Res Clin Oncol. 2022 Jan 30;148(4):803–21. doi: 10.1007/s00432-021-03885-z (PMC8930900; doi:10.1007/s00432-021-03885-z)

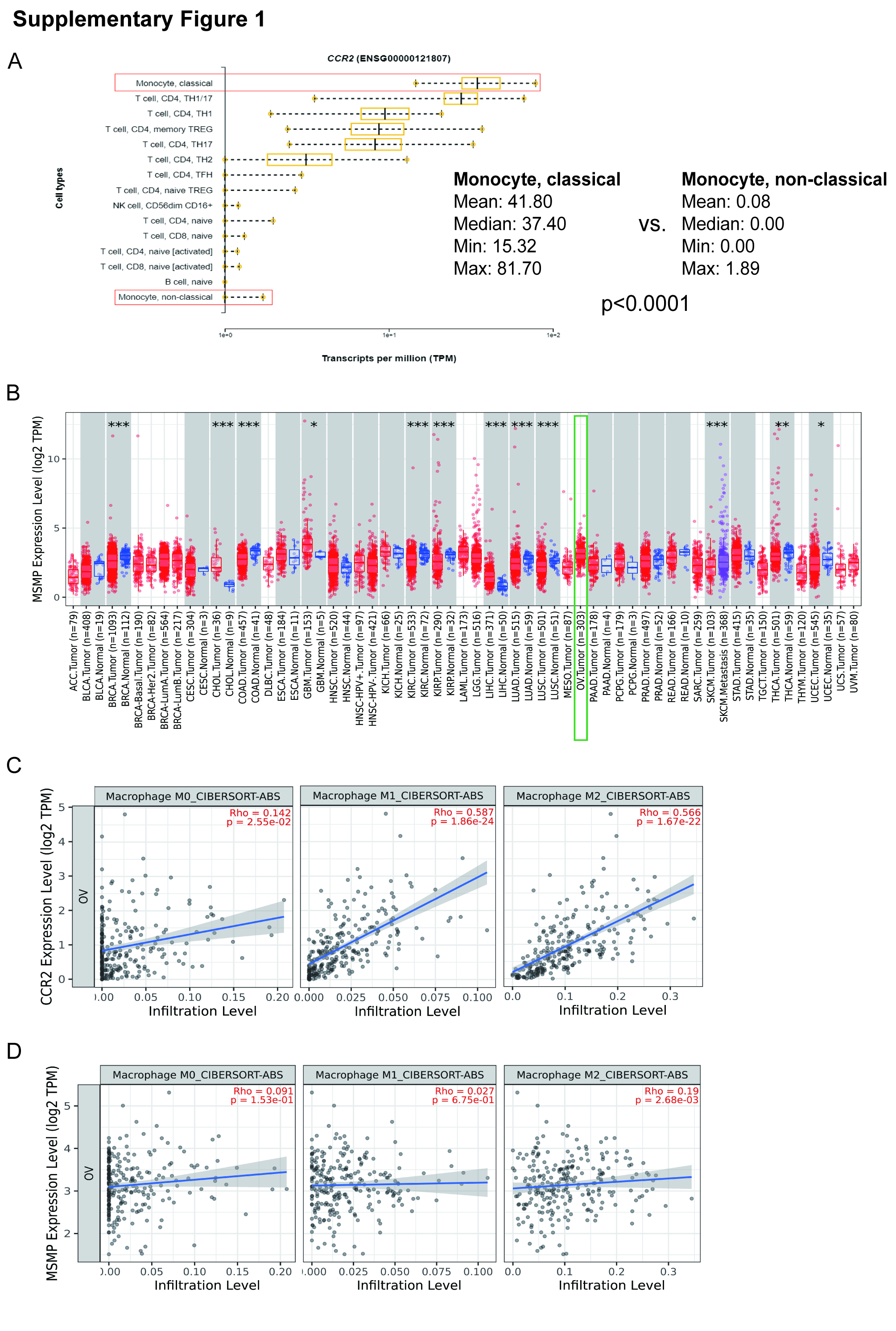

Supplement: Supplementary file 2 — Supplementary file2 (TIF 26961 kb) [file 432_2021_3885_MOESM2_ESM.tif]

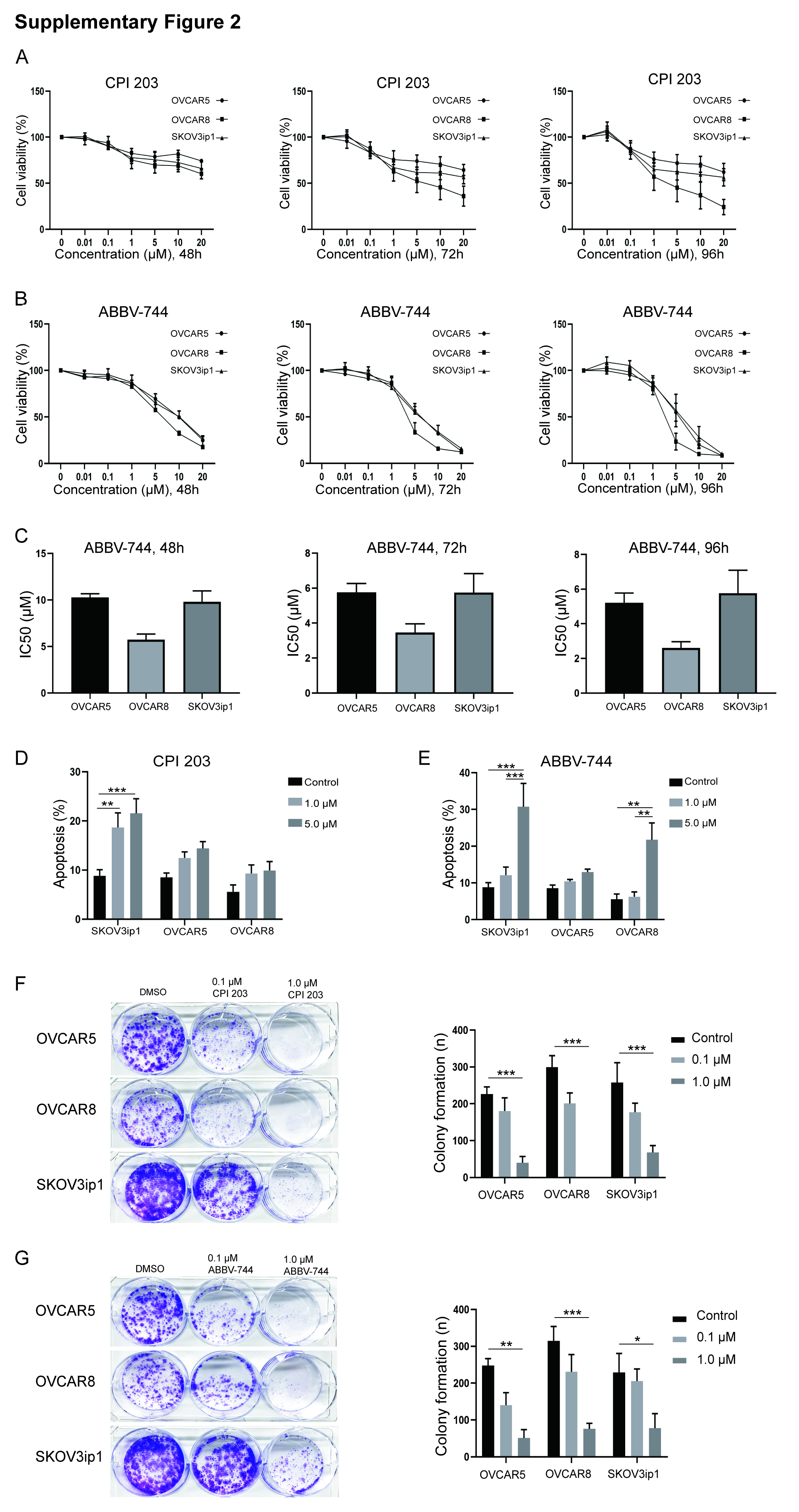

Supplement: Supplementary file 3 — Supplementary file3 (TIF 33522 kb) [file 432_2021_3885_MOESM3_ESM.tif]

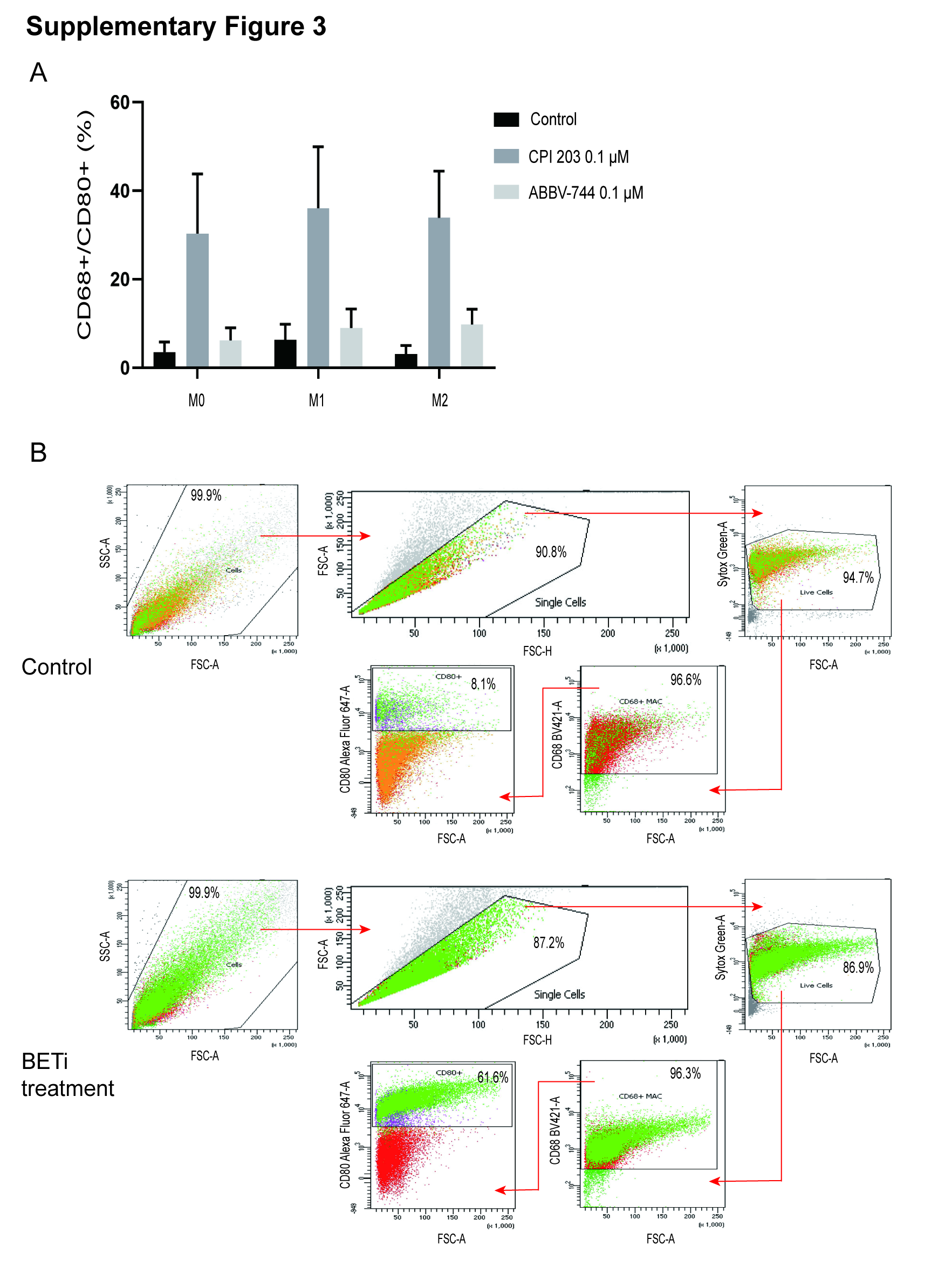

Supplement: Supplementary file 4 — Supplementary file4 (TIF 18253 kb) [file 432_2021_3885_MOESM4_ESM.tif]

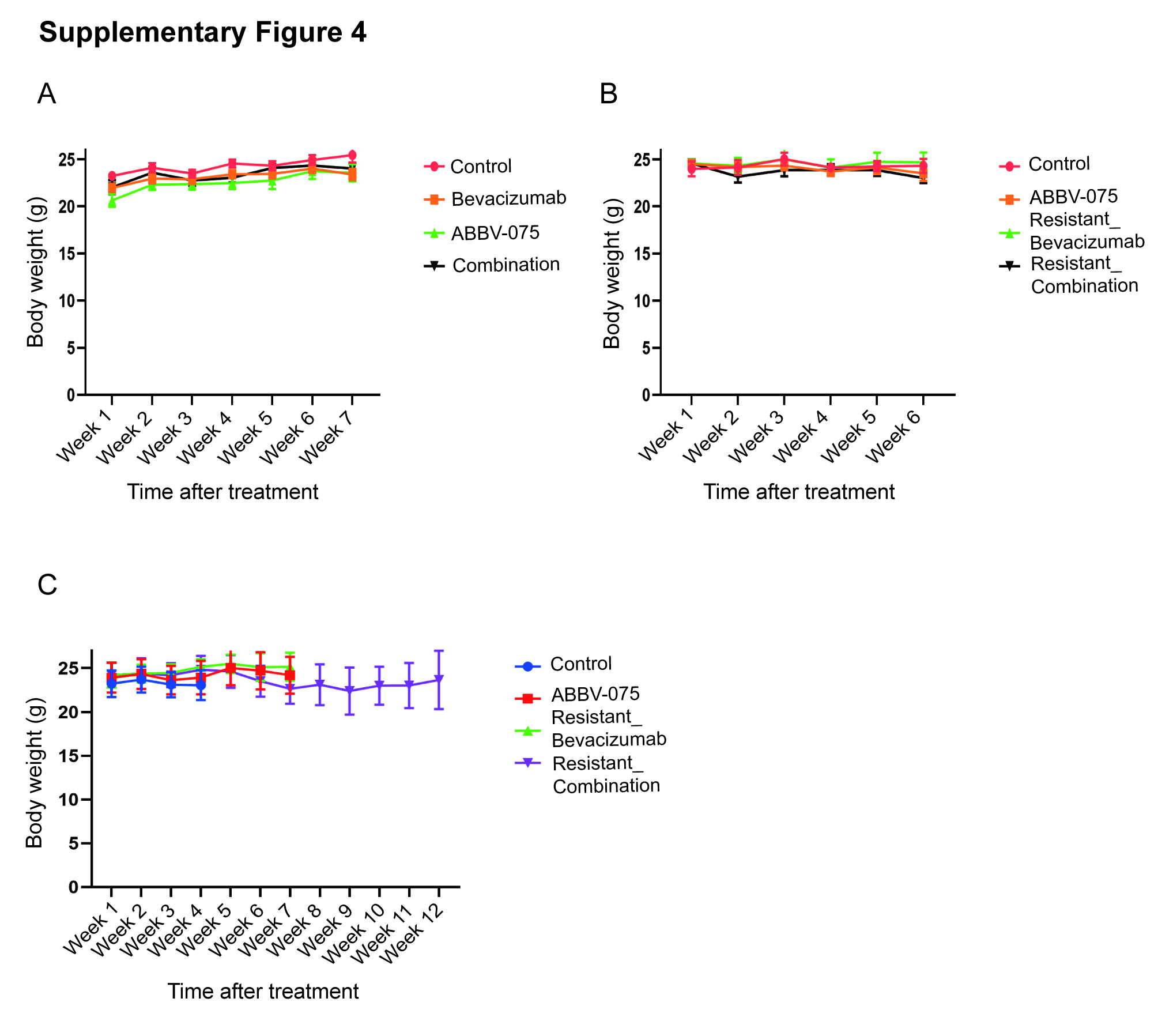

Supplement: Supplementary file 5 — Supplementary file5 (TIF 14688 kb) [file 432_2021_3885_MOESM5_ESM.tif]
